# Presenting Symptoms and Antibiotic Timing Among Hospitalized Adults With Presumed Sepsis

**DOI:** 10.1001/jamanetworkopen.2026.35575

**Published:** 2026-09-28

**Authors:** Anna Conlon, Sarah Seelye, Emily Walzl, Harsh Bhanderi, Ashwin B. Gupta, Jennifer K. Horowitz, Namita Jayaprakash, Nicholas Kuhl, Anurag N. Malani, Elizabeth McLaughlin, Pat Posa, Claire Shappell, Stephanie Parks Taylor, Scott A. Flanders, Hallie C. Prescott, Elizabeth S. Munroe

**Affiliations:** 1 Department of Medicine, University of Michigan, Ann Arbor; 2 VA Center for Clinical Management Research, Ann Arbor, Michigan; 3 Department of Emergency Medicine, Emory University and Emory Healthcare, Atlanta, Georgia; 4 Corewell Health, Grand Rapids, Michigan; 5 Division of Infectious Disease, Department of Medicine, Trinity Health, Ann Arbor, Michigan; 6 Division of Pulmonary and Critical Care, Brigham and Women’s Hospital, Boston, Massachusetts; 7 Department of Pulmonary and Critical Care, Intermountain Health, Salt Lake City, Utah

## Abstract

**Importance:**

Timely antibiotics are associated with reduced sepsis mortality, particularly among patients with hypotension; however, antibiotic delays are common in practice. Presenting symptoms may influence antibiotic timing and confound the association between antibiotic timing and outcomes.

**Objectives:**

To evaluate whether any presenting symptoms are associated with antibiotic timing and to assess risk-adjusted 30-day mortality by antibiotic timing after adjustment for symptoms.

**Design, Setting, and Participants:**

This cohort study was conducted among adults hospitalized for community-onset presumed bacterial sepsis at 67 hospitals in the Michigan Medicine Safety Consortium from November 2020 to May 2024.

**Exposures:**

Presenting symptoms; time from hospital presentation to first antibiotic administration.

**Main Outcomes and Measures:**

Timely antibiotics, defined as antibiotic administration within 3 hours of hospital presentation for patients with hypotension and within 5 hours for patients without hypotension, was the primary outcome. Secondary outcomes included time to antibiotics, measured as a continuous variable, and 30-day mortality.

**Results:**

Among 29 647 patients included in this analysis, the median (IQR) age was 71 (61-80) years, 14 676 (49.5%) were male, and the median (IQR) Charlson Comorbidity Index was 3 (1-5). Among 6704 patients (22.6%) presenting with hypotension, 4413 (65.8%) received timely antibiotics (within 3 hours). Among 22 942 (77.4%) patients presenting without hypotension, 17 544 (76.5%) received timely antibiotics (within 5 hours). In multivariable analyses, presenting symptoms were independently associated with antibiotic timing. Among patients with and without hypotension, prehospital fever was associated with increased odds of timely antibiotics (with hypotension: adjusted odds ratio [aOR], 1.34 [95% CI, 1.19 to 1.52]; without hypotension: aOR, 1.26 [95% CI, 1.17 to 1.35]), while gastrointestinal symptoms were associated with decreased odds of timely antibiotics (with hypotension: aOR, 0.84 [95% CI, 0.75 to 0.94]; without hypotension: aOR, 0.80 [95% CI, 0.75 to 0.86]). Urinary and respiratory symptoms were associated with antibiotic timing for patients without hypotension (urinary symptoms: aOR, 1.13 [95% CI, 1.05 to 1.22]; respiratory symptoms: aOR, 0.91 [95% CI, 0.85 to 0.99]). Timely antibiotics were associated with reduced 30-day mortality (adjusted absolute difference, −2.10 [95% CI, −3.01 to −1.10] percentage points) among the overall cohort, after adjustment for presenting symptoms.

**Conclusions:**

In this cohort study of 29 647 adults presenting with community-onset presumed bacterial sepsis, presenting symptoms were associated with timing of antibiotic therapy. Even after adjusting for presenting symptoms, timely antibiotics remained associated with lower mortality. These findings suggest that proactive screening for sepsis among patients with less obvious clinical presentations may promote timely recognition and treatment.

## Key Points

**Question:**

Are presenting symptoms associated with antibiotic timing among patients presenting with sepsis?

**Findings:**

In this cohort study of 29 647 adults presenting with community-onset presumed bacterial sepsis, presenting symptoms, including prehospital fever and gastrointestinal symptoms, had significant associations with antibiotic timing after adjustment for illness severity and chronic conditions. Timely antibiotics were associated with significantly reduced 30-day mortality after adjustment for patient characteristics including presenting symptoms.

**Meaning:**

In this study, presenting symptoms were associated with antibiotic timing, and timely treatment remained associated with lower mortality, even after adjustment for presenting symptoms.

## Teaser

This cohort study evaluates whether any presenting symptoms are associated with timely antibiotic prescription among adults with community-onset presumed bacterial sepsis in Michigan.

## Introduction

Sepsis is a common cause of hospitalization that contributes to up to half of in-hospital deaths in the United States.^1,2^ Prompt initiation of antibiotic therapy is associated with improved survival,^3,4,5,6,7^ with greater absolute benefit among patients in shock.^3,8,9^ Due to the challenge of conducting randomized trials of time-sensitive treatments like antibiotics,^10,11^ much of the available data about optimal antibiotic timing in sepsis come from observational studies, which provide a large body of evidence supporting the benefit of timely antibiotic initiation.^5,6,12^ The 2026 Surviving Sepsis Campaign (SSC) guidelines stratify antibiotic timing recommendations based on likelihood of infection and presence of shock,^13^ recommending that antibiotic therapy be initiated as soon as possible, ideally within 1 hour, when sepsis is definite or probable or when sepsis is possible and shock is present. The guidelines suggest rapid evaluation of infectious vs noninfectious causes of illness when sepsis is possible and shock is absent, with initiation of antibiotics within 3 hours if concern for bacterial infection persists.^13^ However, despite these guidelines, treatment delays are common in practice.^14,15^

The timeliness of antibiotic initiation is influenced by multiple factors, with prior studies identifying several patient characteristics contributing to earlier antibiotic administration (eg, higher illness severity, older age, increased comorbidity burden^9,16,17^) or later antibiotic administration (eg, acute kidney dysfunction, liver dysfunction^9^). Antibiotic timing additionally varies across hospitals, even after adjustment for differences in patient case mix.^14,18,19^ However, while prior studies have identified patient and hospital characteristics associated with antibiotic timing, few have assessed whether presenting symptoms affect antibiotic timing, as these data are rarely captured in structured datasets.^20^ This is an important gap, as clinicians rely on presenting symptoms to diagnose infection and make decisions about antibiotic initiation. Furthermore, presenting symptoms have been suggested as a key potential confounder not addressed in most prior observational studies showing the benefit of timely antibiotics.^20,21^

In this study, we examined the association between presenting symptoms and antibiotic timing among patients hospitalized with community-onset sepsis. We additionally assessed risk-adjusted 30-day mortality by antibiotic timing after adjustment for presenting symptoms and other patient characteristics.

## Methods

### Setting

This was a cohort study of patients with community-onset sepsis hospitalized November 2020 through May 2024 at 67 hospitals participating in the Michigan Hospital Medicine Safety Consortium (HMS)–Sepsis initiative. The study was reviewed by the University of Michigan Institutional Review Board (HUM00188852) and deemed not regulated because HMS is a quality improvement initiative. This study adheres to the Strengthening the Reporting of Observational Studies in Epidemiology (STROBE) reporting guidelines.^22^

HMS is a statewide collaborative quality initiative sponsored by Blue Cross Blue Shield of Michigan. Professional abstractors at each hospital collect data on a random sample of adults hospitalized for community-onset sepsis into the centralized HMS-Sepsis registry, as described previously^23^ (eAppendices 1 and 2 in Supplement 1).

Eligible hospitalizations are identified through a 2-step process. First, hospitalizations with a principal discharge diagnosis of sepsis, infection, or respiratory failure are identified. Second, abstractors confirm evidence of both infection and acute organ dysfunction during the first 2 encounter days, similar to the Centers for Disease Control and Prevention’s Adult Sepsis Event (CDC-ASE) surveillance criteria.^24^ This 2-step process is designed to identify patients with confirmed community-onset sepsis while avoiding biases introduced by variable diagnostic sepsis coding over time and across hospitals.^25^ Patients admitted via transfer from another hospital are excluded.

### Study Population

In this study, we included patients in the HMS-Sepsis registry who presented with presumed bacterial sepsis. We excluded patients with positive COVID-19 or influenza test results, and a small number of patients without clinical evidence of sepsis at initial presentation, defined by meeting all 4 of the following criteria: no symptoms of infection; fewer than 2 systemic inflammatory response syndrome criteria during the first 2 hours of hospital presentation; no leukocytosis (ie, initial white blood cell count ≤12 000/µL [to convert to ×10^9^ per liter, multiply by 0.001]); and no lactate elevation (ie, initial lactate ≤18 mg/dL [to convert to millimoles per liter, multiply by 0.111]).

### Subgroups

In the primary analysis, we examined patients presenting with and without hypotension separately, as the magnitude of absolute benefit of timely antibiotics differs among patients with vs without hypotension^9^ and HMS targets for timely antibiotic administration differ by presenting blood pressure, as described later. We defined hypotension as systolic blood pressure less than 90 mm Hg, mean arterial pressure less than 65 mm Hg, or initiation of intravenous vasopressor therapy (ie, norepinephrine, vasopressin, epinephrine, phenylephrine, dopamine, or angiotensin II) within 2 hours of presentation. We required only a single hypotensive blood pressure measurement, as even nonsustained hypotension confers an increased risk of mortality among patients with sepsis.^26^

### Data Collection

We used data from the HMS-Sepsis registry. In this registry, presenting symptoms are manually abstracted based on clinician documentation using standardized definitions and grouped into 5 categories: respiratory symptoms, prehospital fever, altered mental status, gastrointestinal symptoms, and urinary symptoms, as described in eTable 1 in Supplement 1. Prehospital fever is defined as documentation of chills, rigors, subjective fever, or reported temperature prior to admission of greater than 99.0 °F. Reported temperatures of 99.0 to 100.4 °F are included to encompass a broad definition of prehospital fever and capture patient-reported low-grade fevers. Antibiotic order and administration timing are recorded from electronic health record time stamps. Thirty-day mortality is obtained for all patients through medical record review, public obituary data, and follow-up by telephone, email, and/or SMS messaging.^27^ Data on hospital sepsis programs were collected in a fall 2023 survey sent to all hospitals participating in HMS-Sepsis.

### Outcomes

The primary outcome was receipt of timely antibiotics, defined as administration of a qualifying systemic antibiotic within 3 hours of presentation for patients presenting with hypotension or within 5 hours for patients presenting without hypotension. Qualifying systemic antibiotics included any intravenous or oral antibiotic included in the CDC-ASE surveillance definition.^24,28^ The 3- and 5-hour targets are consistent with HMS performance benchmarks and SSC guideline recommendations,^13^ after allowing 2 hours for initial triage and evaluation. Secondary outcomes included time to antibiotics, measured as a continuous variable, and 30-day mortality.

### Statistical Analysis

Results were considered statistically significant if the 95% CIs did not cross 0. We considered *P* < .05 (2-sided) to be statistically significant. Analyses were performed using SAS version 9.4 software (SAS Institute) and StataMP version 16.1 software (StataCorp).

#### Antibiotic Timing

First, we used multivariable hierarchal logistic regression to evaluate the association between patient symptoms and timely antibiotics, adjusting for demographic characteristics, chronic conditions, presenting vital signs, and predicted risk for 30-day mortality. Covariates were prespecified based on clinical expertise and prior literature.^11,13,14,15,16,17,18,19^ eTable 2 in Supplement 1 provides covariates and their functional forms. Predicted 30-day mortality was calculated using the HMS-Sepsis mortality model, a validated physiologic risk-adjustment model developed for use in HMS-Sepsis.^23^ Separate models were fit for patients presenting with and without hypotension and included hospital as a random effect, excluding hospitals with fewer than 10 observations. We examined the association of treating hospital with timely antibiotics using a median odds ratio,^29^ which represents the increased odds of receiving timely antibiotics if moving from a hospital with less timely antibiotic delivery to a hospital with greater timely antibiotic delivery.^30,31^

Second, we evaluated time to antibiotics as a continuous outcome by fitting a single multivariable hierarchical linear regression model adjusted for the same covariates as the primary analysis as well as hypotension within 2 hours of presentation, with hospital included as a random effect. Patients who received their first antibiotic at more than 12 hours were excluded.

#### Mortality

To evaluate the association between timely antibiotics and 30-day mortality, we first fit hierarchical logistic regression models for patients presenting with hypotension, patients presenting without hypotension, and the entire cohort. These models adjusted for predicted 30-day mortality as well as characteristics and symptoms associated with timely antibiotics in either patients with or without hypotension in the antibiotic timing analysis. The functional form of continuous covariates was evaluated by comparing linear specifications with restricted cubic splines and assessing model fit statistics. Hospital was included as a random effect, excluding hospitals with fewer than 10 observations. We estimated predicted probabilities (predictive margins) averaging predicted outcomes over the covariate distribution of the population. Absolute risk differences were estimated as the difference between predictive margins, with 95% CIs derived using the delta method. This approach was applied across all sensitivity analyses.

Second, we examined the association of time to antibiotics (as a continuous measure) with 30-day mortality using restricted cubic spline terms added to logistic regression models adjusted for predicted 30-day mortality and characteristics and symptoms associated with timely antibiotic administration in our initial analysis. We considered 3-, 4-, and 5-knot models using the Harrell approach to assign knots^32^ and selected the best model based on Akaike and bayesian information criteria.^33,34^ In the primary continuous analysis, patients receiving antibiotics more than 12 hours after presentation were excluded to avoid blending effects of long time-to-antibiotic intervals with shorter intervals, which may inflate estimates for the average increase in mortality with antibiotic delays.^21^ We performed a sensitivity analysis including patients with antibiotic initiation greater than 12 hours.

#### Sensitivity Analyses

We performed sensitivity analyses examining the association of antibiotic timing modeled as a dichotomous exposure and mortality. First, we excluded patients who did not receive antibiotics within 12 hours of presentation. Second, we excluded patients with evidence of any viral or fungal infection (eTable 3 in Supplement 1). Third, to isolate confirmed bacterial sepsis, we examined the subgroup of patients with positive bacterial cultures on days −1 to 2 of presentation. Fourth, we included patients without signs of sepsis, or with positive COVID-19 or influenza testing. Fifth, we used inverse probability of treatment weighting (IPTW) for the probability of timely antibiotics and a doubly robust logistic regression model. We also performed subgroup analyses to assess the association of antibiotic timing and mortality among patients with each presenting symptom.

#### Exploratory Analysis

To understand presenting symptoms, we evaluated the association of presenting symptoms with likely source of infection based on culture results and coding^35^ and compared overlaps in presenting symptoms. To evaluate hospital variation, we examined the association between hospital characteristics and timely antibiotics by dividing hospitals into quintiles of antibiotic timing and comparing hospital characteristics and structures of care (eg, presence of sepsis order sets, nurse-driven sepsis protocols) between the top quintile, middle 3 quintiles, and the lowest quintile of antibiotic timing using analysis of variance.

## Results

Among 35 410 patients in the HMS-Sepsis registry during the study period, 29 647 (83.7%) had presumed bacterial sepsis (signs of sepsis on presentation and no positive COVID-19 or influenza testing) and were included in this study. Patients had a median (IQR) age of 71 (61-80) years, 14 676 (49.5%) were male, and the median (IQR) Charlson Comorbidity Index was 3 (1-5) (Table 1). The most common presenting symptoms were respiratory (20 602 [69.5%]), prehospital fever (15 921 [53.7%]), and altered mental status (14 653 [49.4%]). A total of 6705 (22.6%) had hypotension within 2 hours of hospital arrival (Figure 1).

**Table 1.** Patient Characteristics and Presentation

Characteristic	Patients, No. (%)		
	All (N = 29 647)	With hypotension (n = 6705)	Without hypotension (n = 22 942)
**Baseline characteristics**			
Age, median (IQR), y	71 (61-80)	71 (62-80)	71.0 (60-80)
Body mass index, median (IQR)^a^	27.6 (23.0-33.5)	27.1 (22.5-32.8)	27.8 (23.2-33.6)
Sex			
Male	14 676 (49.5)	3308 (49.3)	11 368 (49.6)
Female	14 971 (50.5)	3397 (50.7)	11 574 (50.4)
Admitted from SNF, SAR, LTAC, or AR	4188 (14.1)	1344 (20.0)	2844 (12.4)
Hospitalized in prior 90 d	9898 (33.4)	2699 (40.3)	7199 (31.4)
**Chronic conditions**			
Charlson comorbidity score, median (IQR)	3 (1-5)	3 (2-5)	3 (1-5)
Immunocompromised state^b^	5610 (18.9)	1379 (20.6)	4231 (18.4)
Chronic lung disease^c^	10 169 (34.3)	2384 (35.6)	7785 (33.9)
Diabetes (complicated)	5929 (20.0)	1371 (20.4)	4558 (19.9)
Liver disease (moderate or severe)	934 (3.2)	285 (4.3)	649 (2.8)
Chronic kidney disease (moderate or severe)	10 022 (33.8)	2696 (40.2)	7326 (31.9)
Congestive heart failure	7942 (26.8)	2078 (31.0)	5864 (25.6)
Metastatic solid malignant neoplasm	2253 (7.6)	654 (9.8)	1599 (7.0)
Hematologic malignant neoplasm	942 (3.2)	245 (3.7)	697 (3.0)
**Illness severity on presentation**			
Predicted 30-d mortality	0.12 (0.05-0.27)	0.25 (0.12-0.47)	0.10 (0.05-0.21)
**Symptoms on presentation^d^**			
Prehospital fever	15 921 (53.7)	2996 (44.7)	12 925 (56.3)
Gastrointestinal symptoms	12 539 (42.3)	2890 (43.1)	9649 (42.1)
Altered mental status	14 653 (49.4)	4075 (60.8)	10 578 (46.1)
Urinary symptoms	7714 (26.0)	1623 (24.2)	6091 (26.5)
Respiratory symptoms	20 602 (69.5)	4726 (70.5)	15 876 (69.2)
**Vital signs in first or second hour**			
Temperature <96 °F	1217 (4.1)	683 (10.2)	534 (2.3)
Temperature >100.4 °F	6737 (22.7)	1162 (17.3)	5575 (24.3)
Heart rate >100 beats/min	18 313 (61.8)	3907 (58.3)	14 406 (62.8)
Respiratory rate >20 breaths/min	17 401 (58.7)	4655 (69.4)	12 746 (55.6)
Spo_2_ <91% or using oxygen	14 456 (48.8)	4072 (60.7)	10 384 (45.3)

Abbreviations: AR, acute rehab; LTAC, long-term acute care; SAR, subacute rehab; SNF, skilled nursing facility; Spo_2_, oxygen saturation as measured by pulse oximetry.

^a^ Body mass index calculated as weight in kilograms divided by height in meters squared.

^b^ Immunocompromised state defined as any of the following: diagnosis of AIDS/HIV, receipt of corticosteroids in previous 30 days, receipt of immunosuppressive drug in prior 30 days, congenital immunohumoral or cellular immune deficiency state, or organ transplant.

^c^ Chronic lung diseases include chronic obstructive pulmonary disease, chronic pulmonary disease, or receiving home oxygen.

^d^ Prehospital fever defined as chills, rigors, fever (subjective, no documented temperature), reported temperature of 99.0 to 100.4 °F (37.2 to 38.0 °C), or temperature greater than 100.4 °F (>38 °C). Gastrointestinal symptoms included abdominal pain, nausea, vomiting, and diarrhea. Altered mental status defined as confusion, disorientation, delirium, encephalopathy, not acting like themselves, lethargy, somnolence, obtundation, coma, or delirium tremens. Urinary symptoms included urinary frequency, urinary urgency, dysuria, polyuria, urinary hesitancy, urinary incontinence, costovertebral angle pain or tenderness, suprapubic tenderness, flank pain, change in color or smell of urine, hematuria, urinary retention, postvoid residual greater than 200 cc, cloudy urine, dirty urine, and sediment in urine. Respiratory symptoms included increased work of breathing, cough, chest congestion, sputum production, chest pain, chest tightness, wheezing, dyspnea, shortness of breath, rhinorrhea, crackles, rales, dullness to percussion, egophony, hypoxia, new or worsening fraction of inspired oxygen requirement, rhonchi, accessory muscle use, retractions, and tachypnea.

**Figure 1.**
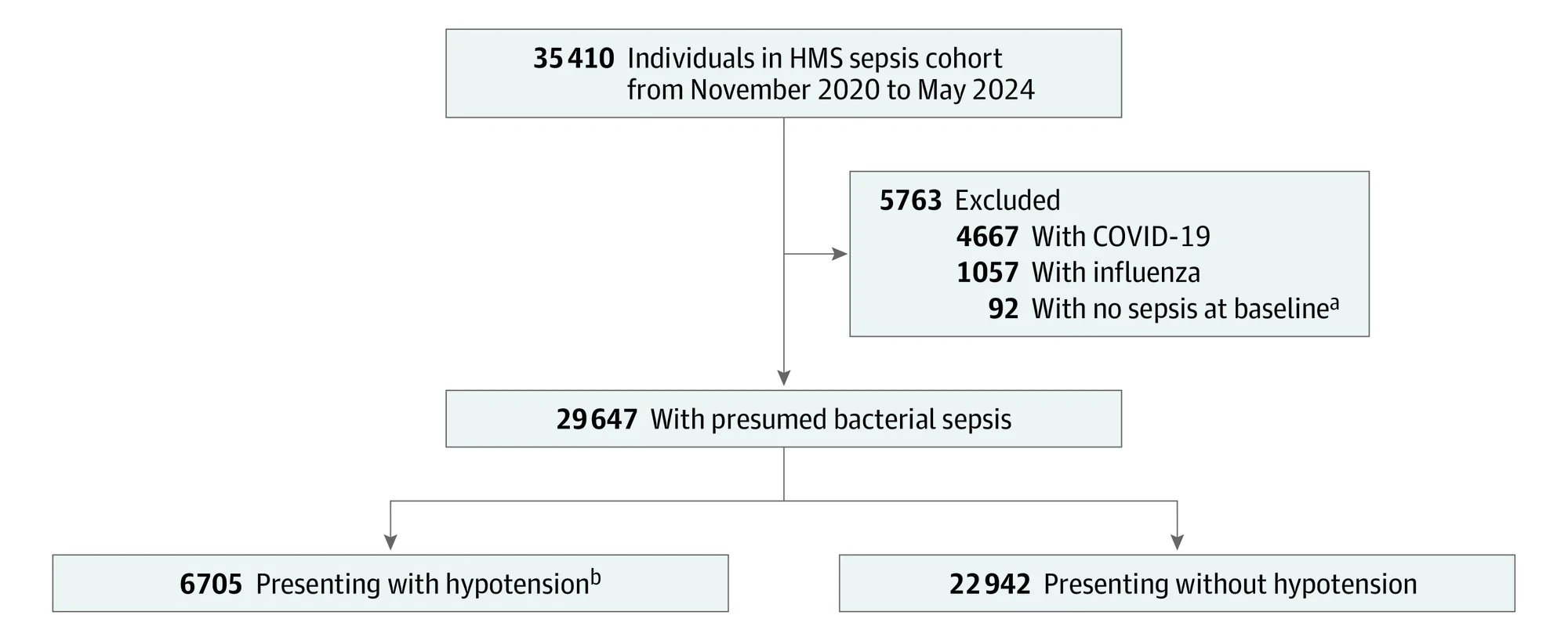
Flowchart of Study Population HMS indicates Michigan Hospital Medicine Safety Consortium. ^a^No clinically documented symptoms of infection on presentation, fewer than 2 systemic inflammatory response syndrome criteria within the first 3 hours of hospital presentation, white blood cell count of 12 000/µL or fewer, and lactate 2 mmol/L or lower on initial laboratory results. To convert white blood cell count to convert to ×10^9^ per liter, multiply by 0.001. ^b^Hypotension within the first 2 hours of hospital presentation was defined by systolic blood pressure less than 90 mm Hg, mean arterial pressure less than 65 mm Hg, or initiation of intravenous vasopressor therapy (ie, norepinephrine, epinephrine, phenylephrine, dopamine, angiotensin II, or vasopressin).

### Patient Symptoms Associated With Timely Antibiotics

Among 6705 patients presenting with hypotension, 4413 (65.8%) received timely antibiotics, defined as antibiotics within 3 hours of presentation. Among 22 942 patients presenting without hypotension, 17 544 (76.5%) received timely antibiotics, defined as antibiotics within 5 hours of presentation. The most common initial antibiotics administered were ceftriaxone, cefepime, and piperacillin-tazobactam (eTable 4 in Supplement 1). The median (IQR) time from hospital presentation to antibiotic delivery was 1.62 (1.07-2.22) hours vs 4.50 (3.57-6.60) hours in the timely vs delayed groups, respectively, for patients presenting with hypotension, and 2.43 (1.60-3.40) hours vs 7.83 (5.83-11.31) hours in the timely vs delayed groups, respectively, for patients presenting without hypotension (eTable 5 in Supplement 1). The proportion of patients receiving timely antibiotics varied across hospitals (eFigure 1 and eFigure 2 in Supplement 1), with nurse-driven sepsis protocols associated with timely antibiotics among patients presenting with hypotension (eTable 6 in Supplement 1). Among patients without hypotension, hospitals with higher rates of timely antibiotics were smaller (<100 beds), were nonmetropolitan, and had lower case-mix index and greater sepsis case volume (eTable 7 in Supplement 1).

In adjusted models for patients with and without hypotension, prehospital fever was associated with increased odds of timely antibiotics (with hypotension: adjusted odds ratio [aOR], 1.34 [95% CI, 1.19 to 1.52]; without hypotension: aOR, 1.26 [95% CI, 1.17 to 1.35]), while gastrointestinal (GI) symptoms were associated with decreased odds of timely antibiotics (with hypotension: aOR, 0.84 [95% CI, 0.75 to 0.94]; without hypotension: aOR, 0.80 [95% CI, 0.75 to 0.86]). Altered mental status was associated with increased odds of timely antibiotics for patients presenting with hypotension (aOR, 1.13 [95% CI, 1.00 to 1.28]) but not in patients without hypotension. In contrast, urinary and respiratory symptoms were associated with antibiotic timing for patients without hypotension (urinary symptoms: aOR, 1.13 [95% CI, 1.05 to 1.22]; respiratory symptoms: aOR, 0.91 [95% CI, 0.85 to 0.99]). (Table 2). Presenting symptoms were generally nonspecific to type or source of infection (eTable 8 and eTable 9 in Supplement 1), with frequent overlap of symptoms (eTable 10 in Supplement 1). In analyses examining antibiotic timing as a continuous variable, hypotension on presentation was associated with a shorter time to antibiotics (−0.54 [95% CI, −0.60 to −0.47] hours) (eTable 11 in Supplement 1).

**Table 2.** Multilevel Multivariable Logistic Regression Model for Receipt of Timely Antibiotics Among Patients With and Without Hypotension^a^

Baseline characteristic	Adjusted odds ratio (95% CI)^b^	
	Patients presenting with hypotension	Patients presenting without hypotension
Age, y^c^	1.00 (1.00-1.01)	1.00 (1.00-1.01)
Body mass index^c^^,^^d^	1.00 (0.99-1.00)	1.00 (0.99-1.00)
Sex		
Male	1.12 (1.01-1.25)	1.04 (0.98-1.11)
Female	1 [Reference]	1 [Reference]
Admitted from SNF, SAR, LTAC, or AR	1.33 (1.14-1.55)	1.29 (1.14-1.44)
Hospitalized in prior 90 d	0.95 (0.85-1.07)	0.99 (0.92-1.07)
Chronic conditions		
Charlson Comorbidity Index	1.00 (0.96-1.05)	1.04 (1.01-1.07)
Immunocompromised status^e^	0.93 (0.81-1.07)	1.01 (0.92-1.10)
Chronic lung disease^f^	1.06 (0.94-1.20)	1.04 (0.97-1.13)
Diabetes (complicated)	1.14 (0.97-1.34)	1.02 (0.93-1.13)
Liver disease (moderate or severe)	0.85 (0.63-1.15)	0.72 (0.59-0.89)
Chronic kidney disease (moderate or severe)	1.03 (0.89-1.19)	0.90 (0.82-0.98)
Congestive heart failure	0.83 (0.73-0.95)	0.87 (0.80-0.95)
Metastatic solid malignant neoplasm	1.10 (0.81-1.50)	0.60 (0.49-0.74)
Hematologic malignant neoplasm	1.04 (0.77-1.41)	0.90 (0.74-1.10)
Illness severity on presentation		
Predicted 30-d mortality	1.56 (1.08-2.26)	1.89 (1.32-2.70)
Vital signs in first or second hour		
Maximum temperature^g^		
<35 °C	1.29 (1.00-1.67)	2.38 (1.72-3.30)
35-36 °C	1.08 (0.87-1.35)	1.01 (0.83-1.23)
36.1-37.8 °C	1 [Reference]	1 [Reference]
37.9-38.0 °C	2.33 (1.52-3.60)	1.89 (1.54-2.33)
38.1-38.3 °C	3.46 (2.35-5.11)	2.46 (2.06-2.93)
38.4-39.9 °C	2.72 (2.18-3.41)	2.85 (2.53-3.21)
Maximum heart rate, beats/min		
<60	1.09 (0.74-1.62)	0.68 (0.52-0.90)
61-90	0.86 (0.74-0.99)	0.70 (0.64-0.77)
91-100	1.01 (0.86-1.19)	0.86 (0.78-0.94)
101-124	1 [Reference]	1 [Reference]
>124	1.14 (0.98-1.33)	1.24 (1.13-1.37)
Maximum respiratory, breaths/min		
<20	1 [Reference]	1 [Reference]
20-21	1.18 (0.98-1.41)	1.21 (1.10-1.32)
22-24	1.31 (1.10-1.54)	1.50 (1.36-1.65)
25-30	1.64 (1.38-1.95)	1.86 (1.67-2.07)
>30	1.86 (1.55-2.24)	1.93 (1.71-2.19)
Spo_2_<91% or using oxygen	1.30 (1.15-1.48)	1.38 (1.27-1.50)
Symptoms on presentation^h^		
Prehospital fever	1.34 (1.19-1.52)	1.26 (1.17-1.35)
Gastrointestinal symptoms	0.84 (0.75-0.94)	0.80 (0.75-0.86)
Altered mental status	1.13 (1.00-1.28)	0.99 (0.92-1.07)
Urinary symptoms	1.02 (0.90-1.17)	1.13 (1.05-1.22)
Respiratory symptoms	0.91 (0.80-1.03)	0.91 (0.85-0.99)
Hospital effect, median OR (95% CI)	1.34 (1.23-1.45)	1.52 (1.39-1.65)

Abbreviations: AR, acute rehab; LTAC, long-term acute care; SAR, subacute rehab; SNF, skilled nursing facility; Spo_2_, oxygen saturation as measured by pulse oximetry.

^a^ Timely antibiotics defined as antibiotic delivery within 3 hours of hospital presentation for hypotensive cohort and within 5 hours of hospital presentation for normotensive cohort.

^b^ Adjusted odds ratio for logistic regression model including hospital as random effect and all variables from the first column as covariates in the model. Hospitals with fewer than 10 observations each were excluded. This included 3 hospitals accounting for 14 patients in the model of patients presenting with hypotension; no hospitals had fewer than 10 observations in the model of patients presenting without hypotension.

^c^ Variables were included in the functional form of a spline (eTable 3 in Supplement 1). Adjusted odds ratios of the continuous variable are presented for interpretability.

^d^ Body mass index calculated as weight in kilograms divided by height in meters squared.

^e^ Immunocompromised status defined as any of the following: AIDS/HIV, receipt of corticosteroids in previous 30 days, receipt of immunosuppressive drug in prior 30 days, congenital immunohumoral or cellular immune deficiency state, or transplant.

^f^ Chronic lung diseases included chronic obstructive pulmonary disease, chronic pulmonary disease, or receiving home oxygen.

^g^ Odds ratios are not reported for temperatures of 40 °C or greater because model did not converge given small number of patients with this maximum temperature.

^h^ Prehospital fever defined as chills, rigors, fever (subjective, no documented temperature), or reported temperature of greater than 99.0 °F. Gastrointestinal symptoms included abdominal pain, nausea, vomiting, and diarrhea; Altered mental status defined as confusion, disorientation, delirium, encephalopathy, not acting like themselves, lethargy, somnolence, obtundation, coma, or delirium tremens. Urinary symptoms included urinary frequency, urinary urgency, dysuria, polyuria, urinary hesitancy, urinary incontinence, costovertebral angle pain or tenderness, suprapubic tenderness, flank pain, change in color or smell of urine, hematuria, urinary retention, postvoid residual greater than 200 cc, cloudy urine, dirty urine, and sediment in urine. Respiratory symptoms included increased work of breathing, cough, chest congestion, sputum production, chest pain, chest tightness, wheezing, dyspnea, shortness of breath, rhinorrhea, crackles, rales, dullness to percussion, egophony, hypoxia, new or worsening fraction of inspired oxygen requirement, rhonchi, accessory muscle use, retractions, and tachypnea.

### Association of Antibiotic Timing and Mortality

Among patients presenting with hypotension, timely antibiotics modeled as a dichotomous exposure was associated with a numeric reduction in 30-day mortality that was not statistically significant (adjusted mortality, 32.1% vs 32.9%; adjusted absolute difference, −0.84 [95% CI, −3.00 to 1.32] percentage points). Among patients presenting without hypotension and among the combined group of patients with and without hypotension, timely antibiotics were associated with reduced 30-day mortality (without hypotension: adjusted mortality, 15.2% vs 17.3%; adjusted absolute difference, −2.18 [95% CI, −3.26 to −1.10] percentage points; combined: adjusted mortality, 18.9% vs 21.0%; adjusted absolute difference, −2.10 [95% CI, −3.01 to −1.10] percentage points). While there was evidence of nonlinearity of continuous covariates in these models, results were unchanged by using restricted cubic spline forms, thus linear forms were retained for ease of interpretation. Findings were consistent across all sensitivity analyses (Table 3) and in subgroup analyses by presenting symptom (eTable 12 in Supplement 1). In the sensitivity analysis using IPTW, covariates were well-balanced after weighting (eFigure 3 in Supplement 1), with results similar to primary analysis (Table 3).

**Table 3.** Association Between Timely Antibiotics and 30-Day Mortality Among the Primary, Secondary, and Sensitivity Analysis Populations

Population	Patients in cohort, No.	Adjusted 30-d mortality (95% CI), %^a^		Difference (95% CI), percentage points^a^	aOR of 30-d mortality (95% CI)^a^	*P* value
		Patients receiving timely antibiotics (95% CI)	Patients receiving delayed antibiotics (95% CI)			
Presumed bacterial sepsis (primary analysis population)						
All	29 647	18.9 (18.2 to 19.7)	21.0 (19.9 to 22.0)	−2.10 (−3.01 to −1.10)	0.85 (0.79 to 0.92)	<.001
With hypotension	6705	32.1 (30.3 to 33.8)	32.9 (30.7 to 35.0)	−0.84 (−3.00 to 1.32)	0.95 (0.83 to 1.08)	.45
Without hypotension	22 942	15.2 (14.5 to 15.8)	17.3 (16.3 to 18.4)	−2.18 (−3.26 to −1.10)	0.82 (0.74 to 0.90)	<.001
Sensitivity analysis 1						
Excluding patients who did not receive antibiotics within 12 h	28 032	19.1 (18.3 to 19.8)	20.5 (19.4 to 21.7)	−1.46 (−2.50 to −0.43)	0.89 (0.81 to 0.96)	.005
Sensitivity analysis 2						
Excluding patients with positive viral or fungal testing^b^	28 941	19.1 (18.3 to 19.9)	21.1 (20.1 to 22.2)	−2.00 (−2.98 to −1.04)	0.85 (0.79 to 0.92)	<.001
Sensitivity analysis 3						
Confirmed bacterial sepsis	12 262	19.7 (18.7 to 20.7)	22.1 (20.6 to 23.7)	−2.44 (−3.98 to −0.90)	0.82 (0.73 to 0.93)	.002
Sensitivity analysis 4						
All patients in the HMS-Sepsis registry, including patients with positive viral testing and patients without signs of sepsis	35 410	19.0 (18.2 to 19.8)	20.8 (19.8 to 21.8)	−1.80 (−2.64 to −0.97)	0.86 (0.81 to 0.92)	<.001
Sensitivity analysis 5						
Primary analysis population, using inverse probability of treatment weighting for probability of timely antibiotics and doubly robust logistic regression	29 647	18.9 (18.0 to 19.7)	20.7 (19.5 to 21.9)	−1.81 (−3.07 to −0.55)	0.86 (0.78 to 0.95)	.004

Abbreviations: aOR, adjusted odds ratio; HMS, Michigan Hospital Medicine Safety Consortium.

^a^ Multilevel model with hospital as random effect, adjusted for predicted mortality score; factors significantly associated with timely antibiotics among patients with or without hypotension, including admission from a facility, Charlson Comorbidity Index, liver disease, chronic kidney disease, congestive heart failure or ejection fraction less than 40%, metastatic solid malignant neoplasm, maximum temperature in first 2 hours, maximum heart rate in first 2 hours, maximum respiratory rate in first 2 hours, oxygen saturation as measured by pulse oximetry less than 91% or using oxygen; and presenting symptoms, including prehospital fever, gastrointestinal symptoms, altered mental status, urinary symptoms, and respiratory symptoms. Hospitals with fewer than 10 observations each were excluded. (This included 3 hospitals accounting for 14 patients in the model of patients presenting with hypotension; no hospitals had <10 observations in the model of patients presenting without hypotension.) Predicted probabilities (ie, predictive margins) were estimated by averaging predicted outcomes over the covariate distribution of each population. Absolute risk differences were estimated as the difference between predictive margins, with 95% CIs derived using the delta method. This approach was applied across all sensitivity analyses.

^b^ eTable 4 in Supplement 1 includes details of definitions of viral and fungal infection.

In adjusted logistic regression models with time to antibiotics modeled using restricted cubic spline terms with 3 knots placed at the 10th, 50th, and 90th percentiles of the observed time to antibiotics (eTable 13 in Supplement 1), longer time to antibiotic delivery was associated with increased adjusted 30-day mortality, with a steeper slope over the first 6 hours among patients presenting with hypotension compared with those presenting without hypotension (Figure 2). Findings were similar in the sensitivity analysis that included patients with time to antibiotics greater than 12 hours (eFigure 4 in Supplement 1).

**Figure 2.**
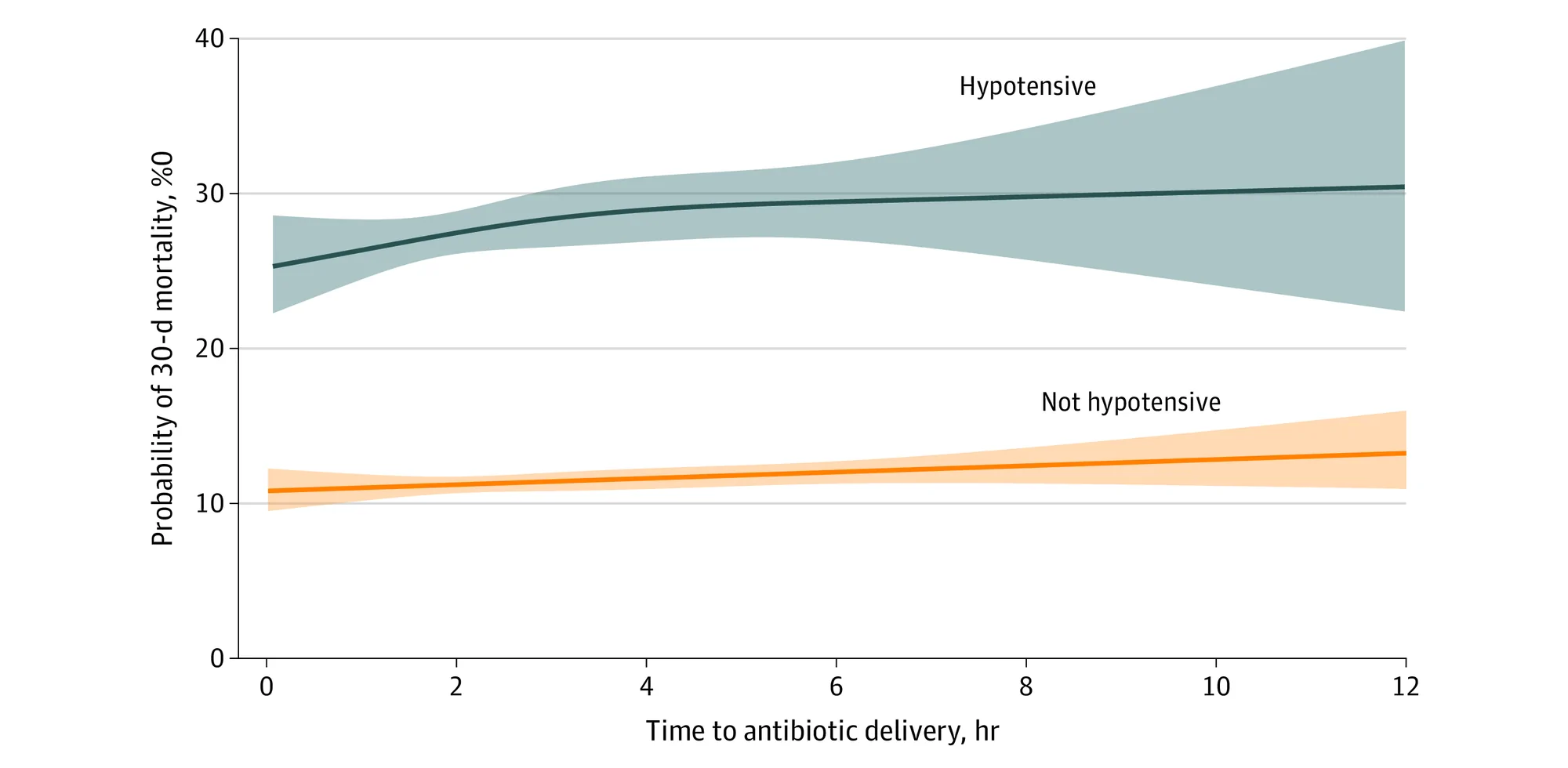
Line Graph of Adjusted 30-Day Mortality Among Adult Patients With Community-Onset Presumed Bacterial Sepsis, With and Without Hypotension This visualization of the spline regression model shows the association of time-to-antibiotics with 30-day mortality using restricted cubic spline terms added to adjusted logistic regression models. Patients with a time to first antibiotic of greater than 12 hours were excluded. Shaded areas indicate the 95% CIs. Time to antibiotics was included as a 3-knot spline term, as this yielded lower Akaike and bayesian information criterion values than 4-knot or 5-knot models, as shown in eTable 13 in Supplement 1. Knots correspond to 1.0, 2.7. and 6.2 hours from hospital presentation.

## Discussion

In this multihospital cohort study of nearly 30 000 patients with community-onset presumed bacterial sepsis, presenting symptoms were associated with antibiotic timing. Particularly, among both patients presenting with and without hypotension, reported fever was associated with timely antibiotics while GI symptoms were associated with delayed antibiotics. Urinary and respiratory symptoms were additionally associated with antibiotic timing for patients presenting without hypotension. Timely antibiotics remained associated with reduced 30-day mortality even after accounting for presenting symptoms.

Early sepsis treatment depends on the ability to diagnose infection,^36^ and presenting symptoms are often key to recognizing infection in practice.^37,38,39^ Our study uniquely captured data about presenting symptoms, which were obtained from clinician notes by professional abstractors using a standardized approach. We found that among both patients with and without hypotension, prehospital fever (reported temperature >99.0 °F or symptoms of fever prior to presentation) was associated with timely antibiotics. Notably, reported prehospital fever or fever symptoms (present in more than 50% of patients) were much more common than having an objective fever (>100.4 °F) in the first 2 hours of emergency department (ED) presentation (23% of patients), underscoring the importance of eliciting history of prehospital fever or fever symptoms rather than relying solely on objective vital signs. In contrast, GI symptoms were associated with delays in antibiotic initiation, possibly due to the wide range of both infectious and noninfectious conditions that can present with GI symptoms. It is important to note that, while many GI infections are not bacterial, GI symptoms are nonspecific and were the presenting symptoms for many types of infections in our cohort, including UTI, pneumonia, and bacteremia, and often overlapped with other presenting symptoms. Interestingly, GI symptoms have also been associated with delayed recognition of other infectious syndromes, such as malaria.^40^ In patients without hypotension, urinary symptoms, which may help identify infection, were associated with timely antibiotics, while respiratory symptoms, which often have broad differential diagnoses, were associated with delayed antibiotics. These findings highlight the importance of a patient’s clinical history in facilitating sepsis identification and treatment and suggest that paying particular attention to patients with symptoms that may mask sepsis, such as GI and respiratory symptoms, may be helpful in early sepsis diagnosis.

Rates of timely antibiotics varied across hospitals, even after adjusting for case mix. Consistent with prior studies, nurse-driven ED sepsis protocols were associated with timely antibiotics among patients with hypotension, where earlier recognition and treatment are key.^41,42^ Yet, while most hospitals in our collaborative reported having standardized sepsis order sets and guidelines, fewer than half reported using nurse-driven ED protocols, highlighting a potential target for future quality improvement efforts.

Consistent with prior studies, we found that timely antibiotics were associated with improved 30-day mortality in our cohort of patients with presumed bacterial community-onset sepsis.^3,4,5,6,7,43^ While many prior studies have assessed the association between antibiotic timing and outcomes in patients with sepsis,^5,6,9,12,16,43,44,45,46,47^ most have been unable to control for patients’ presenting symptoms, which are potential confounders of the association between timely antibiotics and mortality, as patients without clear infectious symptoms may receive delayed antibiotics while also having greater underlying illness severity, leading to higher mortality risk.^21^ Recent research has attempted to address this shortcoming by controlling for presenting symptoms in evaluating the association between Severe Sepsis and Septic Shock Management Bundle (SEP-1) adherence and mortality,^7^ showing no reduction in mortality with higher SEP-1 adherence after adjustment for presenting symptoms. Importantly, the study by Rhee at al^7^ focused on the association of SEP-1 bundle adherence with mortality. However, the SEP-1 bundle includes additional measures beyond early antibiotic therapy, including lactate measurement and blood culture collection, which may not directly impact mortality^48^ and thus may dilute the mortality benefit of early antibiotics. Our study examined a larger cohort of patients with granular data on presenting symptoms and focused specifically on the association between timely antibiotics and mortality. As expected, we found that patient symptoms were associated with antibiotic timing. Yet, after accounting for these symptoms, other patient characteristics and illness severity, timely antibiotics were still associated with improved mortality, strengthening existing evidence for the benefit of early antibiotics.

While we found a significant association between timely antibiotics and mortality in our overall cohort and in patients without hypotension, we did not observe a statistically significant association among patients with hypotension. We caution that this finding should not be taken to mean that timely antibiotics are not important for patients with hypotension. Numerous prior studies have shown that timely antibiotics are associated with lower mortality in patients with hypotension and/or shock, with a larger absolute benefit in this population than in patients without hypotension.^3,6,9,43^ Our study may have failed to detect a statistically significant association between timely antibiotics and mortality in patients with hypotension due to the timely antibiotic cutoff we chose (3 hours), as most patients in our study with hypotension, even those receiving delayed antibiotics, received antibiotics within a short time frame. We dichotomized antibiotic timing in the primary analysis to align with the performance measures used in our statewide quality improvement initiative. However, biologically, the relationship between time to antibiotics and mortality is likely continuous, without a clear threshold effect. The spline regression models treating time to antibiotics as a continuous variable demonstrated a steep increase in mortality from hours 1 to 6 of antibiotic administration in patients with hypotension, supporting the growing body of evidence that timely antibiotics save lives and current guidelines for rapid initiation of antibiotics for patients presenting with hypotension.^10,37^

### Strengths and Limitations

Strengths of our study include the large, multicenter dataset with a diverse patient population and data abstraction performed by professional abstractors collecting data uniformly using standardized forms, enabling accurate capture of both objective and subjective patient data, such as presenting symptoms. Furthermore, our cohort used a robust definition of sepsis that involves a 2-step process and medical record review to confirm the presence of both infection and acute organ dysfunction.

Our study also has several limitations. As an observational study, there may be residual confounding despite attempts to adjust for multiple prespecified potential confounders, including the HMS predicted mortality model developed and validated in this patient population.^23^ Given our goal to evaluate the magnitude of the effect size of multiple symptoms, we did not correct for multiple comparisons, so the results of secondary associations should be considered exploratory. Additionally, given the large sample size, some patient characteristics meeting statistical significance for an association with timely antibiotics have unclear clinical significance. Our cohort was limited to patients treated in Michigan, which might limit generalizability. However, included hospitals have diverse characteristics representative of many US hospitals. Furthermore, ascertaining accurate data on presenting symptoms is limited to the accuracy and completeness of documentation in the health record.

## Conclusions

In this cohort study of 29 647 adults presenting with community-onset presumed bacterial sepsis, we highlighted the important association between symptoms and antibiotic timing. We demonstrated an association between timely antibiotics and adjusted 30-day mortality even after adjustment for presenting symptoms. These findings suggest that proactive screening for sepsis among patients with less obvious clinical presentations may promote timely recognition and treatment.

## Notes

Supplement 1. **eFigure 1.** Proportion of Community-Onset Sepsis Admissions Presenting With Hypotension That Received Timely Antibiotics (Within 3 Hours), by Hospital **eFigure 2.** Proportion of Community-Onset Sepsis Admissions Presenting Without Hypotension That Received Timely Antibiotics (Within 5 Hours), by Hospital **eFigure 3.** Standardized Mean Differences of Covariates by Timely Antibiotics Pre– and Post–Inverse Probability of Treatment Weighting (IPTW) for the Primary Analysis Population of Presumed Bacterial Sepsis **eFigure 4.** Adjusted 30-Day Mortality by Time to Antibiotics Among Patients Presenting With vs Without Hypotension **eTable 1.** Classification of Symptoms and Signs Documented in the Medical Record **eTable 2.** Covariates and Their Functional Forms for Multivariable Models **eTable 3.** Definitions for Sensitivity Analysis Populations **eTable 4.** Top 20 Most Frequently Ordered Initial Antibiotics, by Hypotension **eTable 5.** Time to Antibiotic Order and Antibiotic Administration in Hours, by Hypotension **eTable 6.** Characteristics of Hospitals Participating in HMS-Sepsis Cohort, by Timely Antibiotic Administration in Patients With Hypotension **eTable 7.** Characteristics of Hospitals Participating in HMS-Sepsis Cohort, by Timely Antibiotic Administration in Patients Without Hypotension **eTable 8.** Distribution of Presenting Symptoms by Site of Infection Among the Entire Cohort of Patients With Community Onset Sepsis **eTable 9.** Distribution of Presenting Symptoms by Positive Culture Results Among the Entire Cohort of Patients With Community Onset Sepsis **eTable 10.** Overlap in the Distribution of Symptoms on Presentation Among the Entire Cohort of Patients With Community Onset Sepsis **eTable 11.** Multivariable Mixed-Effects Linear Regression Model for Time-to-Antibiotics as a Continuous Variable, Among the Entire Cohort of Patients With Community Onset Sepsis **eTable 12.** Associations Between Timely Antibiotics and 30-Day Mortality by Presenting Symptom **eTable 13.** Akaike and Bayesian Information Criterion for Spline Models Evaluating the Association Between Antibiotic Timing and 30-Day Mortality **eAppendix 1.** Michigan Hospital Medicine Safety (HMS) Collaborative: General Overview **eAppendix 2.** Michigan Hospital Medicine Safety (HMS) Collaborative: Sepsis Data Curation Methods and Inclusion and Exclusion Criteria
eReference.

Supplement 2.
Data Sharing Statement
