# Supplementary material for: Presenting Symptoms and Antibiotic Timing Among Hospitalized Adults With Presumed Sepsis
Source: JAMA Netw Open. 2026 Sep 28;9(9):e2635575. doi: 10.1001/jamanetworkopen.2026.35575 (PMC13621253; doi:10.1001/jamanetworkopen.2026.35575)
Supplement: Supplementary file 1 [file jamanetwopen-e2635575-s001.pdf]

Supplemental Online Content

Conlon A, Seelye S, Walzl E, et al. Patient symptoms and antibiotic timing among hospitalized adults presenting with sepsis. *JAMA Netw Open.* 2026;9(9):e2635575.
doi:10.1001/jamanetworkopen.2026.35575

eFigure 1. Proportion of Community-Onset Sepsis Admissions Presenting With Hypotension That Received Timely Antibiotics (Within 3 Hours), by Hospital

eFigure 2. Proportion of Community-Onset Sepsis Admissions Presenting Without Hypotension That Received Timely Antibiotics (Within 5 Hours), by Hospital

eFigure 3. Standardized Mean Differences of Covariates by Timely Antibiotics Pre– and Post–Inverse Probability of Treatment Weighting (IPTW) for the Primary Analysis Population of Presumed Bacterial Sepsis

eFigure 4. Adjusted 30-Day Mortality by Time to Antibiotics Among Patients Presenting With vs Without Hypotension

eTable 1. Classification of Symptoms and Signs Documented in the Medical Record

eTable 2. Covariates and Their Functional Forms for Multivariable Models

eTable 3. Definitions for Sensitivity Analysis Populations

eTable 4. Top 20 Most Frequently Ordered Initial Antibiotics, by Hypotension

eTable 5. Time to Antibiotic Order and Antibiotic Administration in Hours, by Hypotension

eTable 6. Characteristics of Hospitals Participating in HMS-Sepsis Cohort, by Timely Antibiotic Administration in Patients With Hypotension

eTable 7. Characteristics of Hospitals Participating in HMS-Sepsis Cohort, by Timely Antibiotic Administration in Patients Without Hypotension

eTable 8. Distribution of Presenting Symptoms by Site of Infection Among the Entire Cohort of Patients With Community Onset Sepsis

eTable 9. Distribution of Presenting Symptoms by Positive Culture Results Among the Entire Cohort of Patients With Community Onset Sepsis

eTable 10. Overlap in the Distribution of Symptoms on Presentation Among the Entire Cohort of Patients With Community Onset Sepsis

eTable 11. Multivariable Mixed-Effects Linear Regression Model for Time-to-Antibiotics as a Continuous Variable, Among the Entire Cohort of Patients With Community Onset Sepsis

eTable 12. Associations Between Timely Antibiotics and 30-Day Mortality by Presenting Symptom

eTable 13. Akaike and Bayesian Information Criterion for Spline Models Evaluating the Association Between Antibiotic Timing and 30-Day Mortality

© 2026 Conlon A et al. *JAMA Network Open*.

eAppendix 1. Michigan Hospital Medicine Safety (HMS) Collaborative: General Overview

eAppendix 2. Michigan Hospital Medicine Safety (HMS) Collaborative: Sepsis Data Curation Methods and Inclusion and Exclusion Criteria

eReference.

This supplemental material has been provided by the authors to give readers additional information about their work.

© 2026 Conlon A et al. *JAMA Network Open*.

eFigure 1. Proportion of Community-Onset Sepsis Admissions Presenting With Hypotension That Received Timely Antibiotics (Within 3 Hours), by Hospital

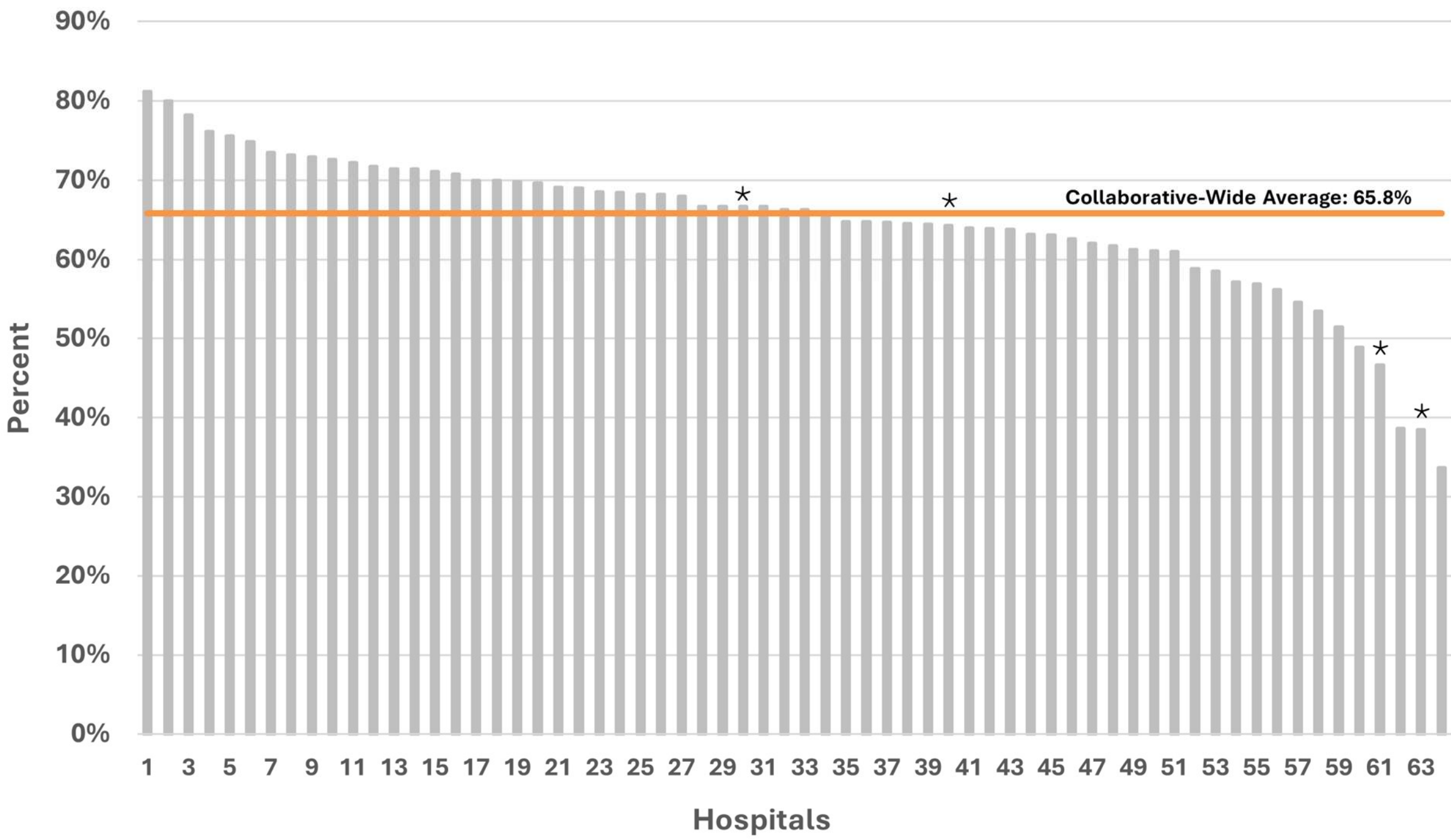

eFigure 1 Legend.
Each bar represents one hospital. The height of the bar reflects the proportion of community-onset sepsis admissions presenting with hypotension that received timely antibiotics. The asterix indicates hospitals with <25 eligible admissions. The horizontal orange line reflects the cohort-wide proportion of admissions with timely antibiotic administration.

© 2026 Conlon A et al. *JAMA Network Open*.

eFigure 2. Proportion of Community-Onset Sepsis Admissions Presenting Without Hypotension That Received Timely Antibiotics (Within 5 Hours), by Hospital

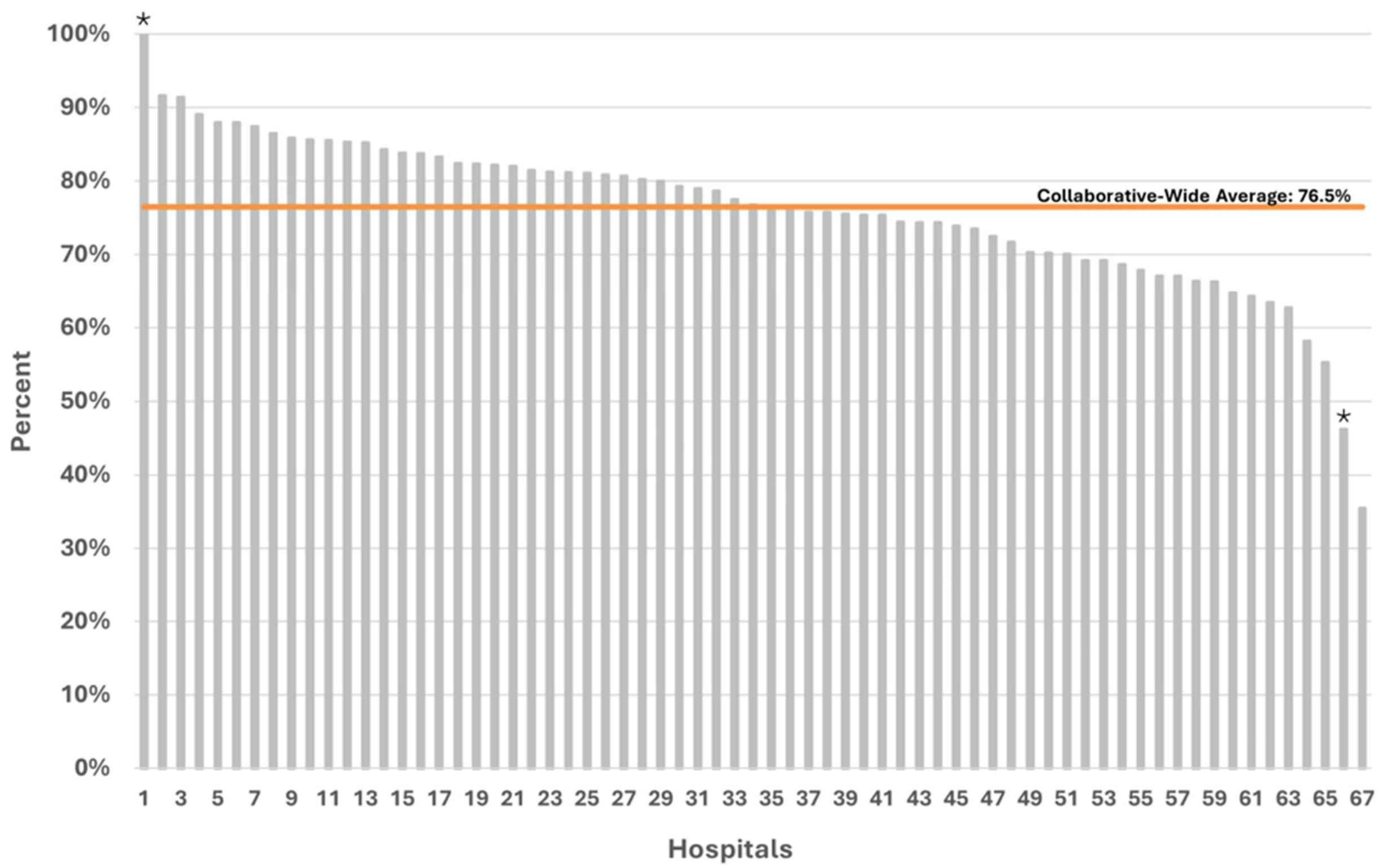

eFigure 2 Legend.
Each bar represents one hospital. The height of the bar reflects the proportion of community-onset sepsis admissions presenting without hypotension that received timely antibiotics. The asterix indicates hospitals with <25 eligible admissions. The horizontal orange line reflects the cohort-wide proportion of admissions with timely antibiotic administration.

© 2026 Conlon A et al. *JAMA Network Open*.

eFigure 3. Standardized Mean Differences of Covariates by Timely Antibiotics Pre– and Post–Inverse Probability of Treatment Weighting (IPTW) for the Primary Analysis Population of Presumed Bacterial Sepsis

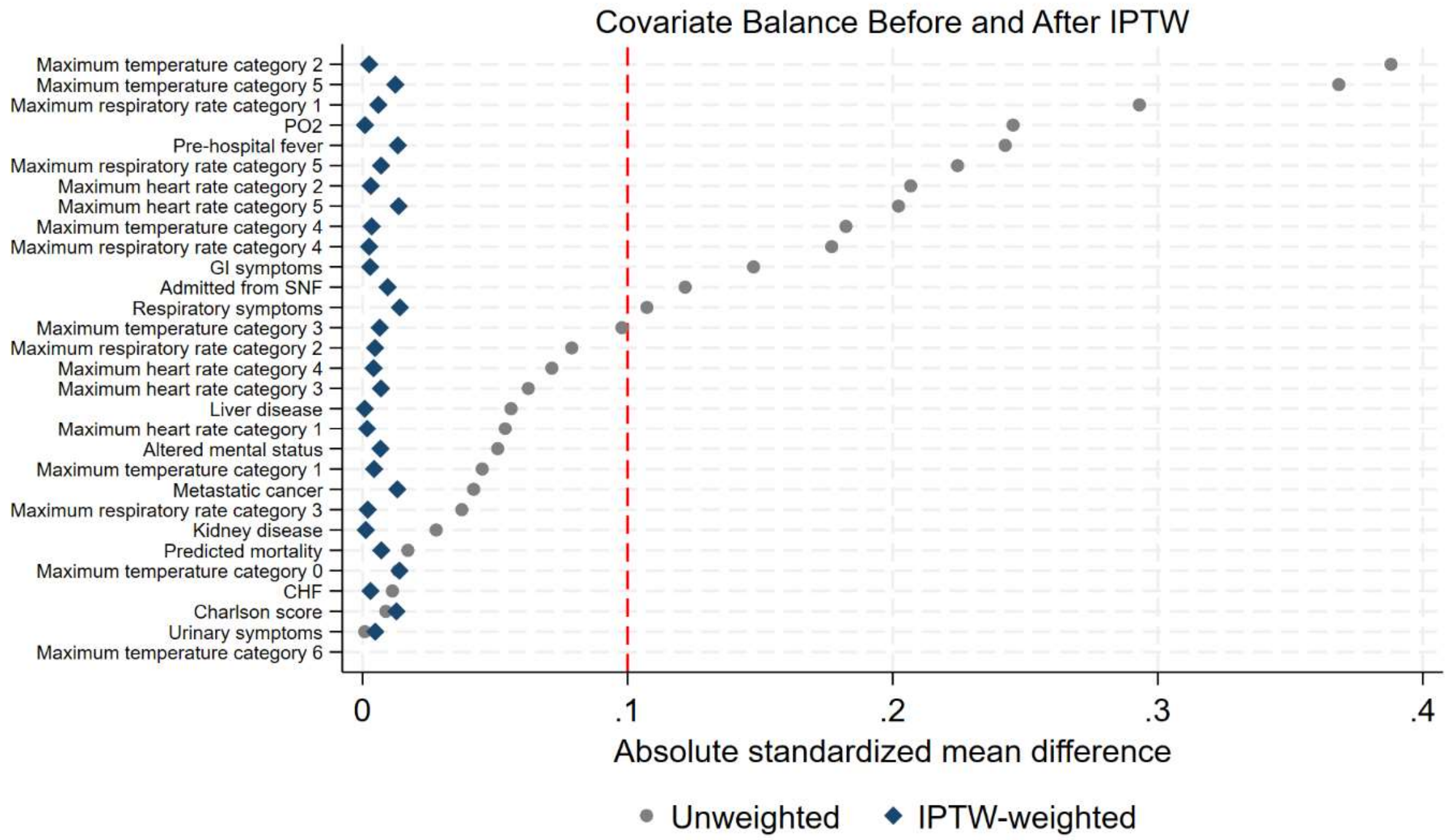

eFigure 3 Legend.
This figure shows the standardized mean differences (x-axis) of the covariates (y-axis) by timely antibiotics that were used in multi-level logistic regression models for 30-day mortality. Each circle represents the raw absolute standardized mean difference of a covariate with respect to timely antibiotics. Each diamond represents the absolute standardized mean difference of a covariate with respect to timely antibiotics after inverse probability of treatment weighting for timely antibiotics.

© 2026 Conlon A et al. *JAMA Network Open*.

eFigure 4. Adjusted 30-Day Mortality by Time to Antibiotics Among Patients Presenting With vs Without Hypotension* (N=29,647)

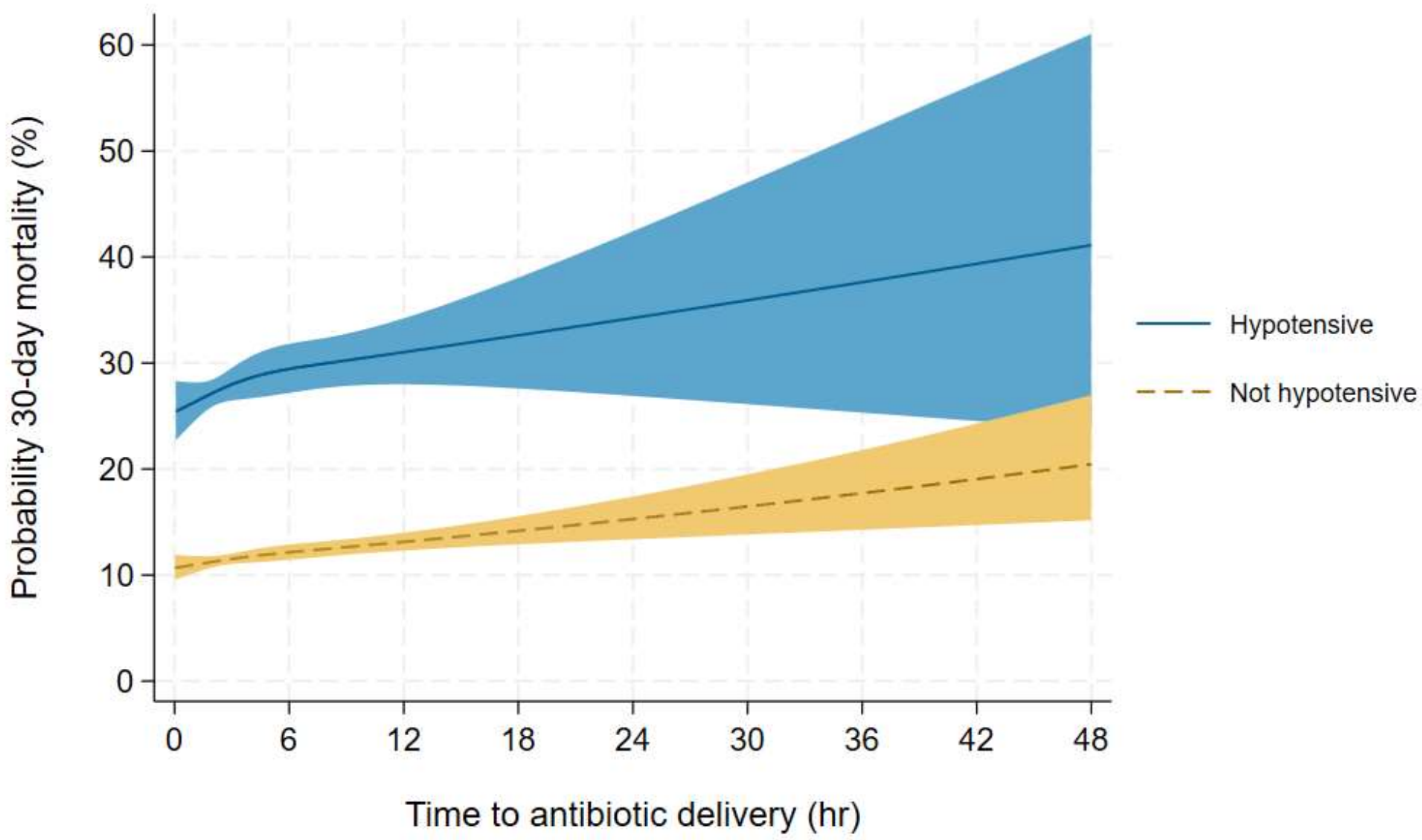

eFigure 4 legend:
This figure shows adjusted 30-day mortality (line) and 95% confidence interval (shaded areas) among patients presenting with hypotension (blue) versus without hypotension (yellow). Time-to-antibiotics was included as a 3-knot spline term, as this yielded lower Akaike and Bayesian information criterion values than 4-knot or 5-knot models, as shown in **eTable 13**. Knots correspond to 1.0, 2.7. and 6.2 hours from hospital presentation.
*Includes patients with presumed bacterial sepsis. Patients who received antibiotics >48 hours following presentation were given a time-to-antibiotics of 48 hours to avoid extreme outliers (31 patients total, 3 hypotensive, 28 without hypotension)

© 2026 Conlon A et al. *JAMA Network Open*.

eTable 1. Classification of Symptoms and Signs Documented in the Medical Record

Domain	Qualifying documentation
Pre-hospital fever	Chills, rigors, fever (Subjective, no documented temperature), reported fever 99.0 – 100.4 F (37.2 to 38 Celsius), reported fever > 100.4 F (> 38 Celsius)
Gastrointestinal symptoms	Abdominal pain, nausea, vomiting, diarrhea
Altered mental status	Altered mental status, confusion, disorientation, delirium, encephalopathy, not acting like themselves, lethargy, somnolence, obtundation, coma, delirium tremens
Urinary symptoms	Urinary frequency, urinary urgency, dysuria, polyuria, urinary hesitancy, urinary incontinence, costovertebral angle pain or tenderness, suprapubic tenderness, flank pain, change in color or smell of urine, hematuria, urinary retention, post void residual > 200cc, cloudy urine, dirty urine, sediment in urine
Respiratory symptoms	Increased work of breathing, cough, chest congestion, sputum production, chest pain, chest tightness, wheezing, dyspnea, shortness of breath, rhinorrhea, crackles, rales, dullness to percussion, egophony, hypoxia, new or worsening Fi02 requirement, rhonchi, Accessory muscle use, retractions, tachypnea

eTable 1 Legend:
Data abstractors review the medical record to determine if any of the qualifying documentation is present in emergency department or hospital admission documentation by physicians or advanced practice providers. Measured vital signs after hospital arrival are not used to determine the presence of subjective symptoms.

© 2026 Conlon A et al. *JAMA Network Open*.

eTable 2. Covariates and Their Functional Forms for Multivariable Models

Variable	Time of measurement	Functional form in models
**Baseline Characteristics**		
Age, years	Presentation	Spline (4 knots)
Male sex at birth	Presentation	Dichotomous
BMI	Presentation	Spline (5 knots)
Admitted from SNF/SAR/LTAC/AR	Presentation	Dichotomous
Hospitalization in the prior 90 days	Presentation	Dichotomous
Charlson Comorbidity Score	Presentation	Linear
Immunocompromised	Presentation	Dichotomous
Chronic lung disease	Presentation	Dichotomous
DM (complicated)	Presentation	Dichotomous
Liver disease (moderate or severe)	Presentation	Dichotomous
CKD (moderate or severe kidney disease)	Presentation	Dichotomous
EF ≤ 40% or history of CHF	Presentation	Dichotomous
Metastatic solid malignancy	Presentation	Dichotomous
Leukemia /lymphoma	Presentation	Dichotomous
**ED Presentation**		
Predicted 30-day mortality from HMS-sepsis model	Calculated using data from first 6 hours	Linear
**Vitals on Presentation**		
Maximum temperature	First 2 hours	Categorical:<br>0 = <35 C<br>1 = 35-36 C<br>2 = 36.1-37.8 C<br>3 = 37.9-38.0 C<br>4 = 38.1-38.3 C<br>5 = 38.4-39.9 C<br>6 = 40.0+ C
Maximum heart rate	First 2 hours	Categorical:<br>1 = <60<br>2 = 61-90<br>3 = 91-100<br>4 = 101-124<br>5 = >124
Maximum respiratory rate	First 3 hours	Categorical<br>1 = Normal (< 20)<br>2 = Abnormal (20- 21)<br>3 = Abnormal (22 - 24)<br>4 = Abnormal (25 - 30)<br>5 = Abnormal (> 30)
Pao2 <91 or on oxygen	First 2 hours	Dichotomous
**Symptoms on Presentation***		
Pre-hospital fever	New or worsening from patient's baseline at time of presentation	Dichotomous
Gastrointestinal symptoms	New or worsening from patient's baseline at time of presentation	Dichotomous
Altered mental status	New or worsening from patient's baseline at time of presentation	Dichotomous
Urinary symptoms	New or worsening from patient's baseline at time of presentation	Dichotomous
Respiratory symptoms	New or worsening from patient's baseline at time of presentation	Dichotomous

*See eTable 1 for qualifying documentation elements for each symptom

© 2026 Conlon A et al. *JAMA Network Open*.

eTable 3. Definitions for Sensitivity Analysis Populations

	**Definitions**
Evidence of viral infection	Any of the following:<br>• Principal ICD10 discharge diagnosis of influenza<br>• Principal ICD10 discharge diagnosis of COVID<br>• Principal ICD10 discharge diagnosis of resp failure AND secondary code for influenza<br>• Positive respiratory virus PCR of days -1 to +2
Evidence of fungal infection	Blood or respiratory cultures positive for any of the following: mycobacterium, Nocardia, pneumocystis, histoplasmosis, aspergillus
Positive bacterial testing	Any patient with positive bacterial testing on days -1 to +2 of the hospital encounter. Positive bacterial testing was defined as positive results on any of the following testing:<br>• Blood culture<br>• Urine culture<br>• Respiratory/sputum culture<br>• Other body fluid culture<br>• Legionella / pneumococcal antigen PCR

© 2026 Conlon A et al. *JAMA Network Open*.

eTable 4. Top 20 Most Frequently Ordered Initial Antibiotics, by Hypotension

	Patients presenting with hypotension, N=6,705		Patients presenting without hypotension, N=22,942	
	**Antibiotic Name**	**N (%)**	**Antibiotic Name**	**N (%)**
1.	Ceftriaxone (Rocephin)	2176 (32.5%)	Ceftriaxone (Rocephin)	10297 (44.9%)
2.	Cefepime (Maxipime)	2038 (30.4%)	Cefepime (Maxipime)	4616 (20.1%)
3.	Piperacillin-tazobactam (Zosyn)	1204 (18.0%)	Piperacillin-tazobactam (Zosyn)	2718 (11.8%)
4.	Vancomycin (Vancocin, Lyphocin)	406 (6.1%)	Azithromycin (Zithromax, Sumamed, Zitrocin)	1337 (5.8%)
5	Azithromycin (Zithromax, Sumamed, Zitrocin)	230 (3.4%)	Vancomycin (Vancocin, Lyphocin)	1096 (4.8%)
6	Metronidazole (Flagyl)	163 (2.4%)	Ampicillin/Sulbactam (Unasyn)	632 (2.8%)
7	Ampicillin/Sulbactam (Unasyn)	120 (1.8%)	Levofloxacin (Levaquin, Quixin)	407 (1.8%)
8	Levofloxacin (Levaquin, Quixin)	75 (1.1%)	Metronidazole (Flagyl)	393 (1.7%)
9	Meropenem (Merrem)	69 (1.0%)	Doxycycline (Doxycycline Hyclate, Doxy, Vibra, Vibramycin)	373 (1.6%)
10	Aztreonam (Azactam)	45 (0.7%)	Cefazolin (Ancef, Kefzol, Zolicef)	190 (0.8%)
11.	Doxycycline (Doxycycline Hyclate, Doxy, Vibra, Vibramycin)	40 (0.6%)	Meropenem (Merrem)	151 (0.7%)
12.	Cefazolin (Ancef, Kefzol, Zolicef)	26 (0.4%)	Aztreonam (Azactam)	138 (0.6%)
13.	Ertapenem (Invanz)	18 (0.3%)	Clindamycin (Cleocin)	96 (0.4%)
14.	Clindamycin (Cleocin)	18 (0.3%)	Ciprofloxacin (Cipro, Ciproxin, Ciprobay)	86 (0.4%)
15.	Ciprofloxacin (Cipro, Ciproxin, Ciprobay)	12 (0.2%)	Ertapenem (Invanz)	69 (0.3%)
16.	Linezolid (Zyvox)	12 (0.2%)	Linezolid (Zyvox)	33 (0.1%)
17.	Piperacillin	5 (0.1%)	Cephalexin (Cefalexin, Keflex, Keftal, Cefanox, Biocef, Panixine	30 (0.1%)
18.	Ampicillin (Omnipen, Principen, Totacillin)	5 (0.1%)	Moxifloxacin (Avelox)	29 (0.1%)
19.	Gentamicin (Gentamycin, Garamycin, Cidomycin, Septopal, Gentamyc	4 (0.1%)	Amoxicillin-clavulanic acid (Augmentin, Co-Amoxiclav)	26 (0.1%)
20.	Daptomycin	4 (0.1%)	Ampicillin (Omnipen, Principen, Totacillin)	25 (0.1%)

© 2026 Conlon A et al. *JAMA Network Open*.

eTable 5. Time to Antibiotic Order and Antibiotic Administration in Hours, by Hypotension

	All Patients N=29,647	Patients presenting with hypotension (N=6,705)		Patients presenting without hypotension (N=22,942)	
		Timely antibiotics (within 3 hours) (N= 4,413)	Delayed antibiotics (greater than 3 hours) (N= 2,292)	Timely antibiotics (within 5 hours) (N= 17,544)	Delayed antibiotics (greater than 5 hours) (N=5,398)
Time to order, hours, median (IQR)	1.98 (1.02-3.52)	0.98 (0.47-1.55)	3.37 (2.57-4.98)	1.70 (0.90-2.60)	5.95 (4.63-9.32)
Time to administration, hours, median (IQR)	2.78 (1.70-4.50)	1.62 (1.07-2.22)	4.50 (3.57-6.60)	2.43 (1.60-3.40)	7.43 (5.83-11.31)

© 2026 Conlon A et al. *JAMA Network Open*.

eTable 6. Characteristics of Hospitals Participating in HMS-Sepsis Cohort, by Timely Antibiotic Administration in Patients With Hypotension

	**Overall N= 66 hospitals**	**Top quantile, N (%)**	**Middle 3 quantiles, N (%)**	**Lowest quantile, N (%)**	**p-value**
Hospital size[1]					
<100 beds	10 (14.9%)	3 (23.1%)	4 (9.8%)	3 (23.1%)	0.054
100-249 beds	18 (26.9%)	5 (38.5%)	10 (24.4%)	3 (23.1%)	
250-499 beds	29 (43.3%)	1 (7.7%)	22 (53.7%)	6 (46.2%)	
500 or more beds	10 (14.9%)	4 (30.8%)	5 (12.2%)	1 (7.7%)	
Urbanicity using RUCC,[2] N(%)					
1 (Metro: population > 1 million)	35 (52.2%)	5 (38.5%)	23 (56.1%)	7 (53.8%)	0.828
2 (Metro: population 250,000 – 1 million)	10 (14.9%)	3 (23.1%)	5 (12.2%)	2 (15.4%)	
3 (Metro: population < 250,000)	10 (14.9%)	2 (15.4%)	7 (17.1%)	1 (7.7%)	
4 (Non-metro: population <20,000)	12 (17.9%)	3 (23.1%)	6 (14.6%)	3 (23.1%)	
Teaching hospital,[3] N(%)	49 (73.1%)	11 (84.6%)	28 (68.3%)	10 (76.9%)	0.550
Case Mix Index,[4] median (IQR)	1.8 (1.7,2)	1.6 (1.5,2.1)	1.8 (1.7,2)	1.9 (1.6,2)	0.773
Sepsis case volume[5]					
≥500 cases per year	23 (34.3%)	5 (38.5%)	12 (29.3%)	6 (46.2%)	
<500 cases per year	44 (65.7%)	8 (61.5%)	29 (70.7%)	7 (53.8%)	0.513
≥ 25% patients at hospital identify as Black	14 (20.9%)	1 (7.7%)	9 (22%)	4 (30.8%)	0.591
Quartile of Area Deprivation Index (ADI)[6]					
Quartile 1 (lowest score)	1.2 (0,3.9)	1.2 (0,2.4)	1.3 (0,3.8)	0 (0,3.9)	0.994
Quartile 2	11.8 (5.4,26.3)	16.7 (2.6,35.6)	12.1 (7.5,26.3)	11.4 (1.9,16.3)	0.476
Quartile 3	27.7 (18.9,37.2)	29.6 (22.5,39.9)	28.8 (18.6,39.5)	23.2 (17.5,27.5)	0.088
Quartile 4 (highest score)	38 (20.1,57.4)	23.6 (16.5,33.7)	38 (24.1,58)	49.8 (44.4,56.9)	0.061
Nonprofit	58 (86.6%)	12 (92.3%)	36 (87.8%)	10 (76.9%)	0.591
**Hospital tools to facilitate timely sepsis treatment (as elicited by spring 2023 survey)**					
Hospital order-set for sepsis	64 (95.5%)	13 (100%)	39 (95.1%)	12 (92.3%)	1.000
Common antibiotics stocked in emergency department	61 (91%)	11 (84.6%)	38 (92.7%)	12 (92.3%)	0.829
Hospital protocol or guideline for sepsis management	59 (88.1%)	11 (84.6%)	36 (87.8%)	12 (92.3%)	1.000
Nurse-driven protocol for sepsis in emergency dept.	30 (44.8%)	9 (69.2%)	13 (31.7%)	8 (61.5%)	0.023
Dedicated sepsis response team	5 (7.5%)	1 (7.7%)	3 (7.3%)	1 (7.7%)	1.000

© 2026 Conlon A et al. *JAMA Network Open*.

[1] Hospital bed size was determined using the 2019 Michigan Certificate of Need Annual Survey, Basic Total Licensed Beds Utilization Statistics. Retrieved 4/21/2021 from https://www.michigan.gov/documents/mdhhs/Report_010_Hospital_Beds_by_HSA_703357_7.pdf
[2] Urbanicity was determined using 2010 US census data and 2013 Rural Urban Continuum Codes (RUCC), collapsed into 4 categories: 1 (Metro- hospitals in counties with >1 million people), 2 (Metro- hospitals in counties with 250,000 to 1 million people), 3 (Metro- hospitals in counties in metro areas with <250,000 people), 4 (RUCC non-metro categories- hospitals in counties with <20,000 people that are adjacent to or not adjacent to a metro area or completely rural). RUCC definitions retrieved 8/5/2023 from https://www.ers.usda.gov/data-products/rural-urban-continuum-codes/
[3] Hospital teaching status were determined based on American Hospital Association definitions. Data were obtained from AMA's Data Hub, retrieved 4/28/2021 from https://guide.prod.iam.aha.org/guide/searchResults
[4] Case Mix Index (CMI) was determined using Centers for Medicare & Medicaid Services Case Mix Index file for Financial Year 2021.
[5] Sepsis case volume based on abstractor volume (by sepsis code only)
[6] Area Deprivation Index, divided into quartiles. Lower scores correlate with lower deprivation. Highest score correlates to higher deprivation.

© 2026 Conlon A et al. *JAMA Network Open*.

eTable 7. Characteristics of Hospitals Participating in HMS-Sepsis Cohort, by Timely Antibiotic Administration in Patients Without Hypotension

	Overall N= 66 hospitals	Top quantile, N (%)	Middle 3 quintiles, N (%)	Lowest quintile, N (%)	p-value
Hospital size[1]					
<100 beds	10 (14.9%)	7 (53.8%)	2 (4.9%)	1 (7.7%)	<.0001
100-249 beds	18 (26.9%)	5 (38.5%)	8 (19.5%)	5 (38.5%)	
250-499 beds	29 (43.3%)	1 (7.7%)	25 (61%)	3 (23.1%)	
500 or more beds	10 (14.9%)	0 (0%)	6 (14.6%)	4 (30.8%)	
Urbanicity using RUCC,[2] N(%)					
1 (Metro: population > 1 million)	35 (52.2%)	4 (30.8%)	23 (56.1%)	8 (61.5%)	0.024
2 (Metro: population 250,000 – 1 million)	10 (14.9%)		7 (17.1%)	3 (23.1%)	
3 (Metro: population < 250,000)	10 (14.9%)	3 (23.1%)	7 (17.1%)		
4 (Non-metro: population <20,000)	12 (17.9%)	6 (46.2%)	4 (9.8%)	2 (15.4%)	
Teaching hospital,[3] N (%)	49 (73.1%)	9 (69.2%)	31 (75.6%)	9 (69.2%)	0.793
Case Mix Index,[4] median (IQR)	1.8 (1.7,2)	1.6 (1.5,1.6)	1.9 (1.7,2)	2 (1.8,2.3)	<.0001
Sepsis case volume[5]					
≥500 cases per year	23 (34.3%)	8 (61.5%)	9 (22%)	6 (46.2%)	0.021
<500 cases per year	44 (65.7%)	5 (38.5%)	32 (78%)	7 (53.8%)	
≥ 25% patients at hospital identify as Black	14 (20.9%)	0 (0%)	10 (24.4%)	4 (30.8%)	0.355
Quartile of Area Deprivation Index (ADI)[6]					
Quartile 1 (lowest score)	1.2 (0,3.9)	0 (0,0.2)	1.7 (0.2,7.4)	1.2 (0,3)	0.065
Quartile 2	11.8 (5.4,26.3)	2.2 (0,16.7)	12.1 (7.5,26.3)	16.3 (6.5,29.3)	0.157
Quartile 3	27.7 (18.9,37.2)	36.1 (22.5,42.9)	27.7 (20.5,35.1)	23.2 (18.6,34.6)	0.551
Quartile 4 (highest score)	38 (20.1,57.4)	43.8 (24.5,60.9)	38 (19.9,53.4)	44.4 (19.4,59.9)	0.785
Nonprofit	58 (86.6%)	13 (100%)	34 (82.9%)	11 (84.6%)	0.355
**Hospital tools to facilitate timely sepsis treatment (as elicited by spring 2023 survey)**					
Hospital order-set for sepsis	64 (95.5%)	13 (100%)	39 (95.1%)	12 (92.3%)	1.000
Common antibiotics stocked in emergency department	61 (91%)	13 (100%)	37 (90.2%)	11 (84.6%)	0.417
Hospital protocol or guideline for sepsis management	59 (88.1%)	12 (92.3%)	35 (85.4%)	12 (92.3%)	1.000
Nurse-driven protocol for sepsis in emergency dept.	30 (44.8%)	9 (69.2%)	16 (39%)	5 (38.5%)	0.176
Dedicated sepsis response team	5 (7.5%)	0 (0%)	4 (9.8%)	1 (7.7%)	0.814

© 2026 Conlon A et al. *JAMA Network Open*.

[1] Hospital bed size was determined using the 2019 Michigan Certificate of Need Annual Survey, Basic Total Licensed Beds Utilization Statistics. Retrieved 4/21/2021 from https://www.michigan.gov/documents/mdhhs/Report_010_Hospital_Beds_by_HSA_703357_7.pdf
[2] Urbanicity was determined using 2010 US census data and 2013 Rural Urban Continuum Codes (RUCC), collapsed into 4 categories: 1 (Metro- hospitals in counties with >1 million people), 2 (Metro- hospitals in counties with 250,000 to 1 million people), 3 (Metro- hospitals in counties in metro areas with <250,000 people), 4 (RUCC non-metro categories- hospitals in counties with <20,000 people that are adjacent to or not adjacent to a metro area or completely rural). RUCC definitions retrieved 8/5/2023 from https://www.ers.usda.gov/data-products/rural-urban-continuum-codes/
[3] Hospital teaching status were determined based on American Hospital Association definitions. Data were obtained from AMA's Data Hub, retrieved 4/28/2021 from https://guide.prod.iam.aha.org/guide/searchResults
[4]Case Mix Index (CMI) was determined using Centers for Medicare & Medicaid Services Case Mix Index file for Financial Year 2021.
[5]Sepsis case volume based on abstractor volume (by sepsis code only)
[6] Area Deprivation Index, divided into quartiles. Lower scores correlate with lower deprivation. Highest score correlates to higher deprivation

© 2026 Conlon A et al. *JAMA Network Open*.

eTable 8. Distribution of Presenting Symptoms by Site of Infection Among the Entire Cohort of Patients With Community Onset Sepsis (N=29,647)

	Site of Infection*									
**Presenting symptom**	**Number of Patients**	**Cardiac**	**Gastrointestinal**	**Genitourinary**	**Respiratory**	**Central nervous system**	**Skin/soft tissue**	**Ear, Nose, & Throat**	**Bacteremia**	**Not identified**
Total Cohort, N(%)	29,647	201 (0.7%)	2,402 (8.1%)	6,735 (22.7%)	12,250 (41.1%)	211 (0.7%)	3,071 (10.3%)	94 (0.3%)	25 (0.1%)	4,658 (15.7%)
Pre-hospital fever, N(%)	15,921	132 (0.8%)	1,313 (8.3%)	3,779 (23.7%)	6,446 (40.5%)	141 (0.9%)	1,862 (11.7%)	58 (0.4%)	12 (0.1%)	2,178 (13.7%)
Gastrointestinal symptoms, N(%)	12,539	82 (0.7%)	1,822 (14.5%)	3,151 (25.1%)	4,104 (32.7%)	81 (0.7%)	1,067 (8.5%)	45 (0.4%)	7 (0.1%)	2,180 (17.4%)
Altered mental status, N(%)	14,653	117 (0.8%)	1,082 (7.4%)	3,924 (26.8%)	5,456 (37.2%)	173 (1.2%)	1,268 (8.7%)	47 (0.3%)	12 (0.1%)	2,574 (17.6%)
Urinary symptoms, N(%)	7,714	47 (0.6%)	634 (8.2%)	3,553 (46.1%)	1,952 (25.3%)	70 (0.9%)	550 (7.1%)	11 (0.1%)	7 (0.1%)	890 (11.5%)
Respiratory symptoms, N(%)	20,602	129 (0.6%)	1,308 (6.4%)	3,473 (16.9%)	10,825 (52.5%)	122 (0.6%)	1,601 (7.8%)	67 (0.3%)	13 (0.1%)	3,064 (14.9%)

eTable8 Legend. *Site of infection was classified by Clinical Classifications Software Refined (CCSR) category using diagnostic codes from the admission. There is no CCSR category for bacteremia, so the ICD10 diagnosis code for bacteremia was used (R78.81). This method has been used in our cohort and described previously[1]

© 2026 Conlon A et al. *JAMA Network Open*.

eTable 9. Distribution of Presenting Symptoms by Positive Culture Results Among the Entire Cohort of Patients With Community Onset Sepsis (N=29,647)

**Presenting symptom**	**Number of patients**	**Positive viral PCR**	**Positive blood culture**	**Positive respiratory culture or positive Streptococcus pneumoniae or Legionella pneumonia antigen**	**Positive urine culture**	**Other positive body fluid culture**	**No positive viral or bacterial testing**
Total cohort, N (%)	29,647	979 (3.3%)	5,964 (20.1%)	2,251 (7.6%)	7,011 (23.6%)	240 (0.8%)	16,382 (55.3%)
Pre-hospital fever, N(%)	15,921	558 (3.5%)	3,827 (24.0%)	1,216 (7.6%)	3,968 (24.9%)	125 (0.8%)	8,264 (51.9%)
Gastrointestinal symptoms, N(%)	12,539	316 (2.5%)	2,845 (22.7%)	694 (5.5%)	3,096 (24.7%)	133 (1.1%)	6,849 (54.6%)
Altered mental status, N(%)	14,653	308 (2.2%)	3,308 (22.6%)	1,192 (8.1%)	4,143 (28.3%)	113 (0.8%)	7,430 (50.7%)
Urinary symptoms, N(%)	7,714	130 (1.7%)	2,136 (27.7%)	347 (4.5%)	3,309 (42.9%)	53 (0.7%)	3,130 (40.6%)
Respiratory symptoms, N(%)	20,602	826 (4.0%)	3,809 (18.5%)	2,061 (10.0%)	4,033 (19.6%)	178 (0.9%)	11,804 (57.3%)

eTable 9 Legend. This table displays the frequency of positive viral and/or bacterial testing on encounter day -1 to 2 based on a patient's presenting symptoms (row percentages). Bacterial cultures are only counted as positive if they are positive for a likely pathogenic organism. HMS-Sepsis has developed a comprehensive list of likely pathogenic pathogens by tissue site.

eTable 10. Overlap in the Distribution of Symptoms on Presentation Among the Entire Cohort of Patients With Community Onset Sepsis (N=29,647)

	**Symptoms on Presentation**				
**Symptoms on Presentation**	Pre-hospital fever	Gastrointestinal symptoms	Altered mental status	Urinary symptoms	Respiratory symptoms
Pre-hospital fever, N(%)	15,921 (100%)	7,794 (49.0%)	7,688 (48.3%)	4,985 (31.3%)	11,832 (74.3%)
Gastrointestinal symptoms, N(%)	7,794 (62.2%)	12,539 (100%)	5,710 (45.5%)	4,283 (34.2%)	8,742 (69.7%)
Altered mental status, N(%)	7,688 (52.5%)	5,710 (39.0%)	14,653 (100%)	4,321 (29.5%)	10,534 (71.9%)
Urinary symptoms, N(%)	4,985 (64.6%)	4,283 (55.5%)	4,321 (56.0%)	7,714 (100%)	5,005 (64.9%)
Respiratory symptoms, N(%)	11,832 (57.4%)	8,742 (42.4%)	10,534 (51.1%)	5,005 (24.3%)	20,602 (100%)

© 2026 Conlon A et al. *JAMA Network Open*.

eTable 11. Multivariable Mixed-Effects Linear Regression Model for Time-to-Antibiotics as a Continuous Variable, Among the Entire Cohort of Patients With Community Onset Sepsis (N=28,032*)

	**Overall N (%) or median (IQR)**	**Regression coefficient (hours)****	**95% CI**	**P-value**
**Baseline characteristics**				
Age, years†	71 (61-80)	-0.002	-0.004, -0.0003	0.019
Body mass index, kg/m^2†	27.6 (23.0-33.4)	0.002	-0.0001, 0.005	0.101
Male sex	13861 (49.6%)	-0.116	-0.164, -0.068	<0.001
Admitted from SNF/SAR/LTAC/AR	4017 (14.3%)	-0.225	-0.299, -0.150	<0.001
Hospitalized in prior 90 days	9349 (33.4%)	0.011	-0.042, 0.065	0.675
Chronic conditions				
Charlson comorbidity score	3 (1-5)	-0.020	-0.040, -0.001	0.035
Immunocompromised state¶	5287 (18.9%)	-0.017	-0.082, 0.047	0.595
Chronic lung disease§	9673 (34.5%)	-0.003	-0.057, 0.052	0.919
Diabetes mellitus (complicated)	5590 (19.9%)	-0.024	-0.096, 0.048	0.516
Liver disease (moderate or severe)	879 (3.1%)	0.176	0.026, 0.327	0.021
Chronic Kidney disease (moderate or severe)	9438 (33.7%)	0.069	0.002, 0.136	0.043
Congestive heart failure	7460 (26.6%)	0.091	0.030, 0.153	0.004
Metastatic solid malignancy	2124 (7.6%)	0.202	0.058, 0.346	0.006
Hematologic malignancy	893 (3.2%)	0.123	-0.019, 0.265	0.090
**Illness severity on presentation**				
Predicted 30-day mortality	0.12 (0.05,0.27)	-0.390	-0.599, -0.181	<0.001
**Symptoms on presentation**‖				
Pre-hospital fever	15250 (54.4%)	-0.231	-0.286, -0.176	<0.001
Gastrointestinal symptoms	11806 (42.1%)	0.249	0.199, 0.299	<0.001
Altered mental status	13891 (49.6%)	-0.055	-0.108, -0.002	0.041
Urinary symptoms	7371 (26.3%)	-0.034	-0.090, 0.022	0.238
Respiratory symptoms	19561 (69.8%)	0.170	0.113, 0.228	<0.001
**Hypotension** (SBP< 90, MAP<65 or on vasopressors within 2 hours)	6472 (23.1%)	-0.535	-0.597, -0.474	<0.001
**Vital signs on Presentation (1st or 2nd hour)‡**				
Maximum temperature in first 2 hours				
<35 C	649 (2.3%)	-0.620	-0.786, -0.454	<0.001
35-36 C	1,017 (3.6%)	-0.144	-0.274, -0.013	0.031
36.1-37.8 C	19,416 (69.3%)	Reference value		
37.9-38.0 C	885 (3.2%)	-0.525	-0.662, -0.388	<0.001
38.1-38.3 C	1,514 (5.4%)	-0.801	-0.909, -0.692	<0.001
38.4-39.9 C	4,521 (16..1%)	-0.909	-0.981, -0.837	<0.001
40.0+ C	30 (0.1%)	-1.550	-2.268, -0.831	<0.001
Maximum Heart Rate in first 2 hours				
<60	365 (1.3%)	0.220	0.006, 0.434	0.043
61-90	5,576 (19.9%)	0.275	0.208, 0.343	<0.001
91-100	4,580 (16.3%)	0.153	0.083, 0.223	<0.001
101-124	11,068 (39.5%)	Reference value		
>124	6,443 (23.0%)	-0.201	-0.265, -0.138	<0.001
Maximum Respiratory Rate in first 2 hours				
<20	7,626 (27.2%)	Reference value		
20-21	4,430 (15.8%)	-0.232	-0.307, -0.157	<0.001
22-24	5,351 (19.1%)	-0.453	-0.526, -0.380	<0.001
25-30	5,536 (19.8%)	-0.670	-0.746, -0.594	<0.001
>30	5,089 (18.1%)	-0.800	-0.883, -0.717	<0.001
PO2<91 or on oxygen	13,816 (49.3%)	-0.402	-0.459, -0.345	<0.001
**eTable 11 legend.**				

© 2026 Conlon A et al. *JAMA Network Open*.

*Includes patients with presumed bacterial sepsis, with or without hypotension. Excludes patients with a time to first antibiotic of greater than 12 hours.
**Adjusted regression coefficient for linear regression model including hospital as random effect and all variables from the first column as co-variates in the model. Hospitals with <10 observations each were excluded.
†Variables were included in the functional form of a spline (see **eTable 2**). aORs of the continuous variable are presented for interpretability.
¶Immunocompromised: AIDS/ HIV, steroids in previous 30 days, immunosuppression in prior 30 days, congenital immunohumoral or cellular immune deficiency state, transplant
§Chronic lung disease: COPD, chronic pulmonary disease, on home oxygen
‖**Pre-hospital fever**: Chills, rigors, fever (Subjective, no documented temperature), reported fever 99.0 – 100.4 F (37.2 to 38 Celsius), reported fever > 100.4 F (> 38 Celsius); **Gastrointestinal symptoms**: Abdominal pain, nausea, vomiting, diarrhea; **Altered mental status**: Altered mental status, confusion, disorientation, delirium, encephalopathy, not acting like themselves, lethargy, somnolence, obtundation, coma, delirium tremens; **Urinary symptoms**: Urinary frequency, urinary urgency, dysuria, polyuria, urinary hesitancy, urinary incontinence, costovertebral angle pain or tenderness, suprapubic tenderness, flank pain, change in color or smell of urine, hematuria, urinary retention, post void residual > 200cc, cloudy urine, dirty urine, sediment in urine**; Respiratory symptoms**: Increased work of breathing, cough, chest congestion, sputum production, chest pain, chest tightness, wheezing, dyspnea, shortness of breath, rhinorrhea, crackles, rales, dullness to percussion, egophony, hypoxia, new or worsening Fi02 requirement, rhonchi, Accessory muscle use, retractions, tachypnea

© 2026 Conlon A et al. *JAMA Network Open*.

eTable 12. Associations Between Timely Antibiotics and 30-Day Mortality by Presenting Symptom						
**Presenting symptom**	**N in cohort**	**Adjusted 30-day mortality*, timely antibiotics (95% CI)**	**Adjusted 30-day mortality*, delayed antibiotics (95% CI)**	**Adjusted absolute 30-day mortality difference* (95% CI)**	**aOR of 30-day mortality (95% CI)***	**p-value**
Pre-hospital fever	15,921	12.9% (12.1%, 13.6%)	14.7% (13.4%, 15.9%)	-1.8% (-3.1%, -0.5%)	0.83 (0.73, 0.94)	0.004
Gastrointestinal symptoms	12,539	16.6% (15.7%, 17.5%)	18.0% (16.7%, 19.2%)	-1.4% (-2.7%, -0.1%)	0.88 (0.78, 0.99)	0.038
Altered mental status	14,653	25.8% (24.8%, 26.8%)	29.2% (27.6%, 30.7%)	-3.3 (-5.0%, -1.8%)	0.81 (0.73, 0.89)	<0.001
Urinary symptoms	7,714	14.9% (13.9%, 15.9%)	17.1% (15.5%, 18.8%)	-2.2% (-3.9%, -0.5%)	0.81 (0.69, 0.95)	0.010
Respiratory symptoms	20,602	21.2% (20.3%, 22.0%)	22.7% (21.5%, 24.0%)	-1.5% (-2.7%, -0.3%)	0.89 (0.81, 0.97)	0.012

eTable12 Legend. *Multi-level model with hospital as random effect, adjusted for predicted mortality score and factors significantly associated with timely antibiotics among patients with or without hypotension (Table 2), including admission from a facility, Charlson comorbidity score, liver disease, chronic kidney disease, congestive heart failure or ejection fraction <40%, metastatic solid malignancy, pre-hospital fever, GI symptoms, altered mental status, urinary symptoms, respiratory symptoms, maximum temperature in first 2 hours, maximum heart rate in first 2 hours, maximum respiratory rate in first 2 hours, pO_2<91 or on oxygen. We estimated predicted probabilities (predictive margins) by averaging predicted outcomes over the covariate distribution of each population. Absolute risk differences were estimated as the difference between predictive margins, with 95% confidence intervals derived using the delta method. This approach was applied across all subgroup analyses.

© 2026 Conlon A et al. *JAMA Network Open*.

eTable 13. Akaike and Bayesian Information Criterion for Spline Models Evaluating the Association Between Antibiotic Timing and 30-Day Mortality

	**Akaike information criterion**	**Bayesian information criterion**
3-knot model	21725.6	21972.8
4-knot model	21726.0	21989.7
5-knot model	21728.5	22008.7

© 2026 Conlon A et al. *JAMA Network Open*.

eAppendix 1. Michigan Hospital Medicine Safety (HMS) Collaborative: General Overview

General Overview

The Hospital Medicine Safety Consortium (HMS) is a hospitalist-focused Collaborative Quality Initiative (CQI), launched in 2010 with 16 participating hospitals. As of 2023, this data-driven CQI is composed of 69 hospital partners across the state of Michigan. The goal of HMS is to improve the quality of care for hospitalized medical patients who are at risk for adverse events.

The HMS CQI specifically targets hospitalists. Due to their management of diverse groups of hospitalized patients and close interactions with hospital staff such as sub-specialists, nurses, and pharmacists, hospitalists are ideally positioned to champion quality improvement (QI) efforts with a goal of preventing adverse events in hospitalized patients.

Sponsorship/Overall Funding Model

HMS is one of more than 20 CQIs sponsored by Blue Cross Blue Shield of Michigan (BCBSM) as a part of their Value Partnerships Program. Value Partnerships is a collection of programs from BCBSM, physicians, and hospitals across Michigan that make health care work better for everyone. It's a unique, collaborative model using data collection and sharing of best practices to help health professionals improve patient outcomes.

Value Partnerships improves the quality of care, making it more cost-effective based on the following guiding principles:

- Partnering with providers to deliver clinical programs and aligned provider payments that improve the delivery of high quality, affordable health care
- Designing and executing programs in a customized and collaborative manner, rather than using a one-size-fits-all approach
- Offering a portfolio of interdependent programs that collectively drive outcomes
- Focusing on investments in transformation of care processes
- Recognizing and rewarding the performance of organizations as well as individual providers
- Rewarding improvement to create meaningful incentives for all participants and partner organizations
- Encouraging collaboration among participants

Pay for Performance

BCBSM's Hospital Pay-for-Performance (P4P) program recognizes short term acute-care hospitals for achievements and improvements in quality and population-health management. In 2021, the program will pay hospitals, in aggregate, an additional 5% of statewide inpatient and outpatient operating payments — over $200 million statewide. Specifically related to the CQIs, hospitals can earn up to 40% of their P4P points based on performance. To determine hospital-specific performance, each CQI annually develops a Hospital Performance Index Scorecard to assess a hospitals performance over a given year related to key quality initiatives.

For more detailed information, please visit the BCBSM Value Partnership website.

QI Initiatives

- Intravascular Devices (Peripherally Inserted Central Catheters [PICC] & Midline)
  - Reduce short term PICC use (<6 days)
  - Minimize multi-lumen PICC use (non-ICU)
  - Avoidance of PICC use with GFR <45
  - Reduce use of triple lumen PICCs in ICU population and among patients with active malignancy
- Inpatient Antimicrobial Use
  - Do not treat uncomplicated community acquired pneumonia (CAP) for >5 days
  - Reduce use of inappropriate empiric broad-spectrum antibiotics for patients with uncomplicated CAP
  - Reduce use of antibiotics in patient with asymptomatic bacteriuria (ASB)
- Sepsis

HMS Leadership & Committees

The work of the collaborative is led by a leadership team within the HMS Coordinating Center and two committees: the executive committee and the data, design, and publication (DDP) committee.

HMS Coordinating Center

Michigan Medicine houses the Coordinating Center for HMS and is responsible for collecting and analyzing comprehensive clinical data from the participating hospitals. It uses these analyses to evaluate and understand current

© 2026 Conlon A et al. *JAMA Network Open*.

practice, support the implementation of improvement strategies, and to evaluate change over time. The Coordinating Center supports participants in establishing quality improvement goals and assists them in implementing best practices. Coordinating Center Leaders:

- *Dr. Scott Flanders - HMS Program Director*
  Professor of Internal Medicine, serves as Chief Clinical Strategy Officer for Michigan Medicine, Vice Chair of External Relations in the Department of Internal Medicine and is a hospitalist in the Division of Hospital Medicine.
- *Dr. Vineet Chopra - QI Consultant and PICC/Midline Physician Lead*
  Professor of Medicine, Department of Medicine and Robert W. Schrier Chair of Medicine, University of Colorado, Anschutz Medical Campus.
- *Dr. Tejal Gandhi - QI Consultant and Antimicrobial Physician Lead*
  Associate Professor of Internal Medicine in the Division of Infectious Diseases (ID) within Michigan Medicine. She is the Medical Director of the Antimicrobial Stewardship Program at Michigan Medicine and Chair of the Antimicrobial Subcommittee.
- *Dr. Hallie Prescott - QI Consultant and Sepsis Physician Lead*
  Associate Professor of Internal Medicine in the Division of Pulmonary Critical Care within Michigan Medicine and a Staff Physician at the Ann Arbor Veterans Affairs Medical Center.
- *Elizabeth McLaughlin, MS, RN – Program Manager*
  Research Senior Supervisor in the Division of Hospital Medicine, Department of Internal Medicine within Michigan.

For additional Coordinating Center details, please visit the HMS website.

Executive Committee

The purpose of the executive committee is to determine the mission and vision of the collaborative, review current performance and address consortium operational issues, identify and discuss implementation strategies, and establish the future course. The executive committee meets once per year – usually in the Fall – in person. The committee members consist of Coordinating Center Leadership, BCBSM leadership, implementation scientists/local quality experts, and leaders from member hospitals.

Data, Design, and Publications (DDP) Committee

The purpose of the HMS Data, Design, and Publications (DDP) Committee is to review and approve data collection methods, performance index measures, programming issues, data analysis methods, quality improvement materials, and the scholarly work of the collaborative. Physician Champions and Quality Improvement Leads/Administrators from all hospitals are invited to participate on this Committee. A clinical data abstractor lead also participates in this Committee as a representative for that role in discussions. The DDP meets monthly via teleconference and 10 to 15 members attend regularly.

Consortium Building/Recruitment

Eligible hospitals within the State of Michigan are recruited annually. In 2020, HMS became one of seven required CQIs within the BCBSM Value Partnerships portfolio. If a hospital is eligible for participation in a required CQI and, at the time of recruitment (when the coordinating center reaches out to ask a hospital to join), voluntarily elects not to participate, the hospital forfeits the ability to earn the P4P incentive attributed to the CQI. HMS welcomes approximately 10 hospitals to the consortium each year. The below diagram shows the hospital onboarding since the inception of the collaborative. For a complete list of participating hospitals, please visit our website.

© 2026 Conlon A et al. *JAMA Network Open*.

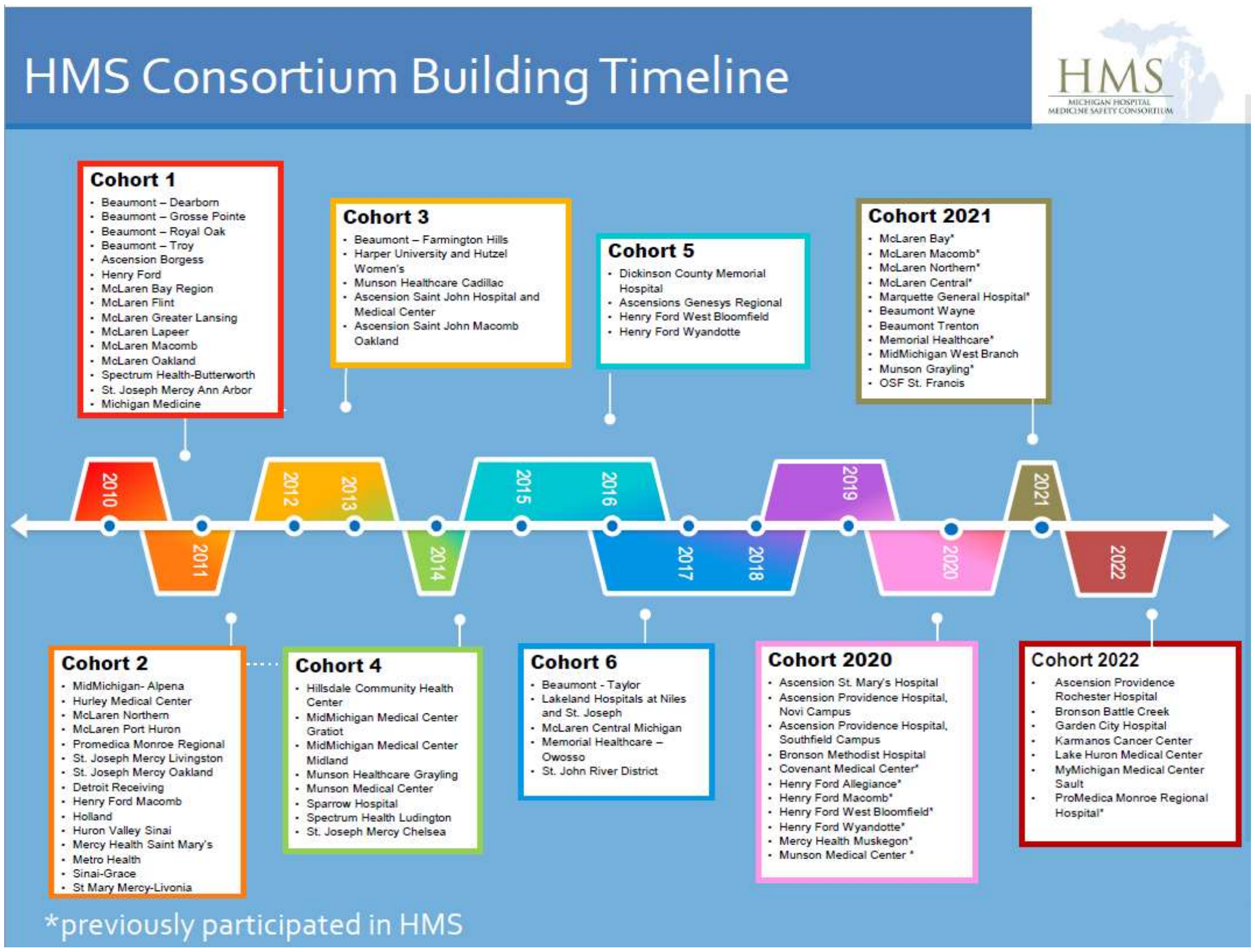

Hospital Participation - Eligibility and Expectations
To participate in HMS, a hospital must meet the following eligibility criteria:

- Be enrolled in BCBSM's Participating Hospital Agreement.
- Have a physician/physician group dedicated to the care of patients in the inpatient hospital setting focused on the care of general medical patients including critical care. For example, hospital program, general internist group, etc.
- Have a sufficient inpatient medical discharge volume of at least 25 eligible adult patients (age 18 years or older) per quarter. Eligibility is based on current QI initiatives. All patients do not have to be managed by the hospitalist program.
- Have organizational commitment for active participation.
- Have an onsite physician champion(s) to act as Clinical Champion.
- Engage members of the multidisciplinary team as it relates to collaborative initiatives. For example, infectious diseases (ID), pharmacy, vascular access, interventional radiology, infection prevention, critical care, etc.
- Have a QI/administrative lead who will work on the collaborative initiatives.
- Have or hire a clinical data abstractor (many of the medical record based data elements to be collected and the follow-up patient calls will require someone with a clinical / healthcare background). While the Collaborative doesn't require a nurse for the role of the clinical data abstractor, it is highly recommended. More than 95% of the clinical data abstractors are nurses.
- Participate in all initiatives in which the hospital is eligible for participation

Participating Hospital Funding Model
Each hospital receives up to 2.0 full time equivalent (FTE) to effectively manage yearly case requirements. Participating sites receive annual FTE funding from BCBSM based on case volumes from the prior year. This payment is intended to cover abstraction costs, attendance at collaborative meetings and a portion of the associated quality improvement time. Case volumes vary year to year, based on initiatives and time of abstraction. FTE amount is based on a percentage of a fully-loaded RN salary of $107,903, which includes both salary and associated benefits.

Participating Hospital- Local Team
Each participating hospital is expected to have a quality lead, clinical data abstractor, and physician champion identified to drive the work of the collaborative at the individual hospital level.

© 2026 Conlon A et al. *JAMA Network Open*.

Quality Lead Expectations

- Administrative contact
- Provide institutional support
- Manage IRB-related issues (if applicable)
- Manage data use agreement
- Hire/assign/troubleshoot data collection staff
- Develop and implement QI efforts
- Participate in site visits

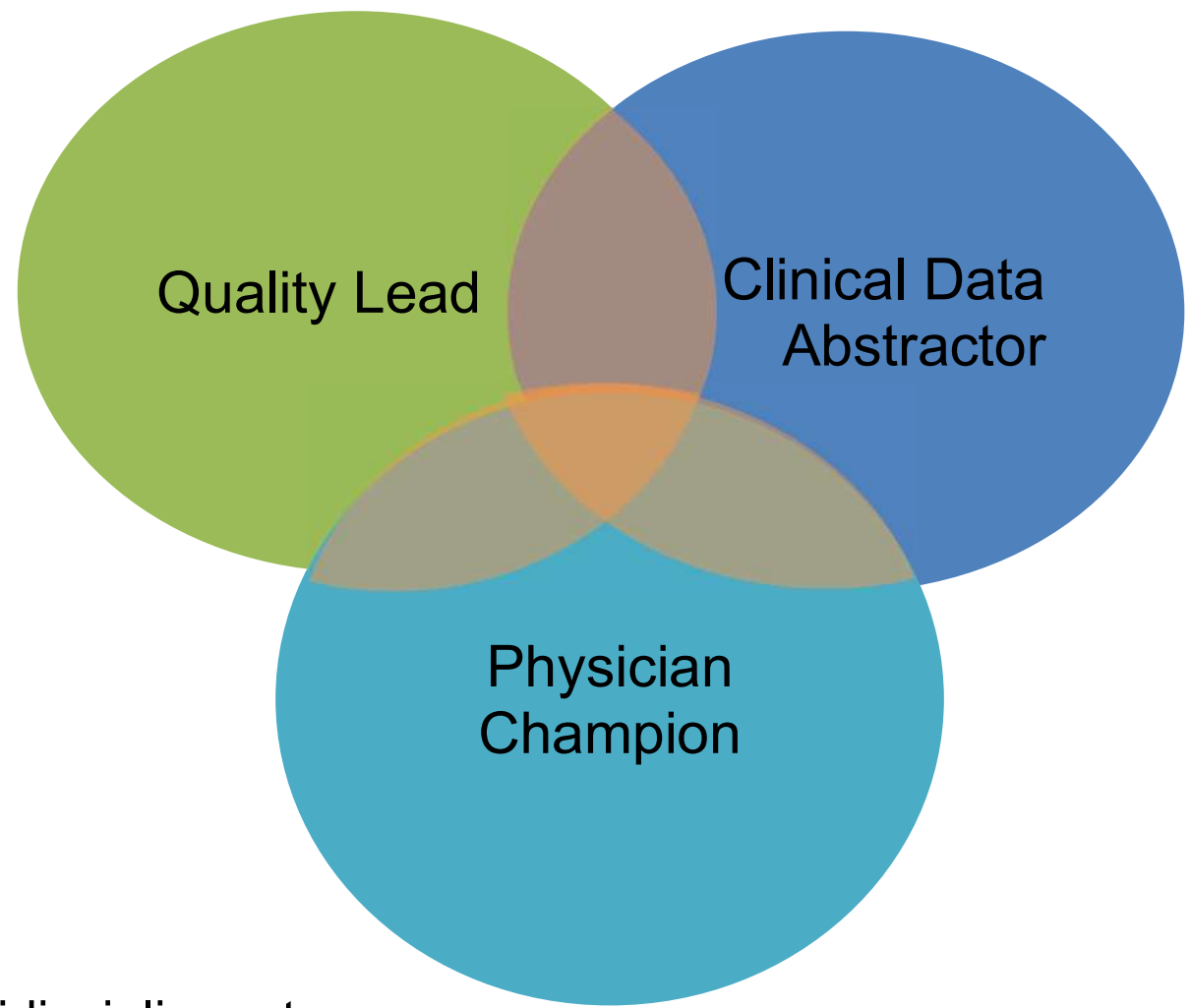

Physician Champion Expectations

- Champion QI efforts
- Develop and implement QI efforts
- Participate in site visits
- Attend Collaborative Wide Meetings
- Facilitate involvement of members across the multidisciplinary team

Clinical Data Abstractor Expectations

- Up to 2.0 FTE could be expected for data collection
- Timely and accurate data collection
- Attend Collaborative Wide Meetings/trainings/conference calls/audits
- Participate in site visits
- Develop and implement QI efforts
- Facilitate completion of semi-annual QI surveys
- Maintain patient lists used for abstraction

As the work of HMS focuses on the hospitalized medical patient, hospitals are encouraged to include members from across the multi-disciplinary medical team in the work of the collaborative. These members include ID physicians, intensivist/critical care, emergency medicine, pharmacy, vascular access, interventional radiology, infection prevention, respiratory therapists, and nursing. Additional departments should be included as necessary.

Tri-annual Collaborative Wide Meetings
Individuals from participating hospitals meet in person three times a year. We encourage hospitals to send their clinical data abstractors, physician champions, and quality leads, as well as other individuals that might be interested. These meetings provide individuals from member hospitals with the opportunity engage with each other in a variety of formats. Each meeting includes a formal discussion of the data from each of the HMS initiatives for the previous quarter, presentations from member hospitals and expert guests, breakout/work group sessions, and networking opportunities. These meetings take place three times per year – in March, July, and November. The July and November meetings are hosted in person; the March meeting is hosted virtually.

At each Collaborative Wide Meeting, attendees are provided with informational reports about their hospital's performance for the previous quarter. These reports provide an in-depth look into the performance of each site on the HMS Performance Measures, as well as other areas applicable to the initiative of interest. For example, our Antimicrobial Use Site Report provides information regarding the top antibiotics administered during the inpatient stay and at discharge, time to de-escalation of broad-spectrum antibiotic coverage, types of physicians ordering urine cultures for ASB patients, and many more items. These reports allow all individuals attending the meeting to gain valuable insights on where their quality improvement efforts should be targeted, and the setting of the collaborative wide meeting allows them to network with other sites who have excelled in those areas to seek ideas on how to improve their performance.

Site Visits
Site visits are conducted at individual participating hospitals either on a voluntary basis or at the discretion of the Coordinating Center. Hospitals may request a site visit with Coordinating Center Leadership and content experts. The site visit includes meetings with hospital leadership, grand round presentations/educational sessions, and focused sessions with local quality teams focused on the collaborative initiatives. The site visits are coordinated and planned in advance by the completion of a questionnaire/initial assessment by the requesting hospital. The assessment is used to determine the

© 2026 Conlon A et al. *JAMA Network Open*.

agenda based on the individual hospital specific improvement needs. As the work of the collaborative is focused on several initiatives the assessment assists with determining the key content experts necessary to be in attendance at the site visit. At the completion of the site visit, a summary is sent to the hospital which includes key findings and additional resources identified during the site visit.

Live Database Reports

Each of the HMS databases are equipped with the ability to view live reports utilizing Business Objects software. These reports provide updated data every 24 hours regarding performance measures (site performance and collaborative performance), fallout case information, demographics, critical/non-critical data errors, completeness of abstracted cases, complication case information (for the PICC/Midline project), and case classification information (for the Antimicrobial Use project).

Individuals who participate in the collaborative either as a clinical data abstractor or a quality administrator have the ability to log into the HMS databases and view these reports at their leisure. The software that HMS utilizes also allows for these reports to be exported as Excel files or PDFs to for hospital specific customization. This information is often utilized at the site level for their committee meetings or presentations to track progress and inform quality improvement efforts at the sites. They also assist the clinical data abstractor to identify errors in their abstraction and resolve them in real time. One of the other uses of these reports is to review individual fallout cases and their clinical scenarios to inform individual clinicians or groups of clinicians of their performance and provide targeted education.

Additional Information

For more information about HMS, please visit the HMS website (http://mi-hms.org/) or follow us on twitter (@HMS_MI). For information related to the CQIs, please visit www.valuepartnerships.com.

© 2026 Conlon A et al. *JAMA Network Open*.

eAppendix 2. Michigan Hospital Medicine Safety (HMS) Collaborative: Sepsis Data Curation Methods and Inclusion and Exclusion Criteria

The HMS Sepsis initiative targets patients diagnosed with community onset Sepsis, or have another qualifying diagnosis, on discharge who meet criteria for organ dysfunction on admission. Of note, the hospital encounter includes emergency, observation, and inpatient time in the hospital.

Data collection for the HMS Sepsis initiative will involve the abstraction of patient data for eligible cases every 2 weeks. The HMS Coordinating Center may notify abstractors periodically of exceptions to this case requirement if the data registry will be unavailable.

Selection of a representative patient sample

A specific sampling process is employed for the project to prevent case selection bias and ensure random selection of eligible cases. The process uses a 14-Day cycle that works as follows. The clinical data abstractor will abstract data on eligible cases from discharge dates covered by a 2-week period. To minimize sampling bias, abstractors are expected to select cases from every day of the cycle being abstracted if possible.

1. This process involves first selecting eligible cases from each day of a cycle, if eligible cases are available.
2. For each day with eligible cases, review the discharge date timestamp [hh:mm] from the hospital encounter.
3. From the timestamp [hh:mm] review, select the first eligible case with the smallest minute value.
    a. For example, if a site has 4 eligible Sepsis cases discharged on the same day at these times: 9:17, 10:27, 12:16 & 21:01, the patient discharged at 21:01 qualifies as the first case for full abstraction for this day, as :01 is the smallest minute value from the list of discharge times of eligible cases on this day. Repeat this same process for selecting the second, third, and subsequent cases for this day. The goal is to have a least one case from each day of the week, if available.

Example for selecting a patient sample:

Discharge Day	**Monday**	**Tuesday**	**Wednesday**	**Thursday**	**Friday**	**Saturday**	**Sunday**
Initial Cases	Patient 1: Discharged at 20:01	Patient 2: Discharged at 17:03	Patient 3: Discharged at 09:01	Patient 4: Discharged at 14:10	Patient 5: Discharged at 09:02	Patient 6: Discharged at 11:13	Patient 7: Discharged at 10:25
Additional Cases (if available)	Patient 8: Discharged at 13:14		Patient 9: Discharged at 08:25	Patient 10: Discharged at 14:45		Patient 11: Discharged at 09:55	

NOTE 1: Hospital encounter includes emergency, observation, and/or inpatient time in the hospital.

NOTE 2: Hospital discharge includes patients discharged alive from inpatient or observation, and patients discharged due to death.

NOTE 3: In the event that the smallest minute value on more than one case is the same, select the case where the patient is discharged later in the day. (For example, if discharge timestamps of 12:10 and 13:10 are both part of the sample, select the case where the patient was discharged at 13:10).

Hospital abstractors are routinely audited by HMS coordinating center staff, and during audits, abstractors are required to demonstrate how they sort admissions and identify eligible hospitalizations according to the above process.

Sepsis Eligibility

Eligible cases will include patients at the time of abstraction who are:

- Patients admitted to a qualifying service (including ICUs) who did not incur a surgical procedure (billed operating room time) within 48 hours of the beginning of the hospital encounter.
    - Patients who went to interventional radiology (IR) or a routine cardiac procedural unit (CPU) within 48 hours of hospital encounter are eligible for inclusion if all other criteria are met.
- A qualifying ICD-10 discharge diagnosis code of sepsis, acute respiratory failure, pneumonia, influenza, or COVID-19. (A separate list was provided)

© 2026 Conlon A et al. *JAMA Network Open*.

    - For cases with a primary code of respiratory failure, a secondary code of pneumonia or influenza must be present.
    - COVID-19 cases also require a secondary discharge diagnosis for organ dysfunction.
- Patient must have received an antibiotic/antiviral/antifungal on Day 1 or Day 2 of the hospital encounter.
    - A primary discharge diagnosis of COVID or any sepsis case that had a positive COVID test within 3 days prior through the first 2 days of the encounter does not require an antibiotic to be administered in order to be eligible for abstraction.
    - Exclude any case where the only antibiotic given to the patient in the first 48 hours was not indicated to treat sepsis/respiratory failure/pneumonia/COVID/influenza or is a chronic/prophylactic antibiotic the patient receives at home.

While no exclusion criteria apply, a stepped inclusion process is noted.

Step 1:
The following patient populations will be excluded via the automated data pull. See the Discharge List Report Specifics for details.

- Patient was pregnant
- Patient under the age of 18
- Patient transferred from another hospital's inpatient or observation unit (ED to ED Transfer is eligible)
    - Please exclude patients who were evaluated in an outside ED and then directly admitted to an inpatient unit at your hospital (without being reevaluated in your on-site ED).
    - Please also exclude patients who transferred from an inpatient rehabilitation unit to an acute care unit.
    - Please exclude patients that were discharged from an inpatient hospitalization within 24 hours prior to presenting for the index admission. This does not include ER visits that do not result in an observation of inpatient admission.
    - Please exclude cases where the patient starts in a procedural suite and is admitted to an inpatient or observation setting without presenting to the ED.

Step 2:
Log the number of cases that were excluded due to surgery occurring within 48 hours of the hospital encounter.

- Do NOT exclude cases in Step 2 who incur an Interventional Radiology (IR) procedure or a routine intervention in a Cardiac Procedure Unit (CPU) within 48 hours of the hospital encounter.
- Exclude cases where there is evidence that the patient is in the Pre-op area by the 48- hour cut off.

Step 3:
Review case for additional exclusions.

- Patient has an order for comfort care/hospice within 3 hours of the beginning of the hospital encounter.
- Patient left against medical advice (AMA) or refused recommended medical care during the hospital encounter.
    - Patient will be ineligible if they have a MI-POST (Michigan Physician Orders for Scope of Treatment) in place.
    - Recommended medical care is defined as the first dose of the initial antibiotics to be given to the patient. This includes COVID cases where the patient refuses the first dose of the recommended anti-infective.
    - Patients who have left AMA during previous encounters remain eligible for abstraction.
- Patient's length of stay for the index hospitalization was greater than 120 days.
- Patient received IV or IM antibiotics in the 24 hours prior to the hospital encounter.
    - ANY IV or IM antibiotics in the 24 hours prior to the hospital encounter would cause the case to be excluded (regardless of location given).
- Patient discharge falls within the 90-day window of a previously recorded/abstracted admission.
- Patients with a primary discharge diagnosis code of COVID with no positive COVID test on Days 1-2 of the encounter or within the three days leading up to the encounter.
- Patients with a primary discharge diagnosis of pneumonia, influenza, COVID, or respiratory failure and who require any amount of home oxygen at baseline.

Step 4:
Complete Organ Function Assessment

© 2026 Conlon A et al. *JAMA Network Open*.

The organ function assessment will evaluate clinical evidence of acute organ dysfunction on Day 1 or 2 of the hospital encounter. Enroll potentially eligible patients and then use organ function calculator to determine eligibility by entering the appropriate lab values and levels of respiratory support administered to the patient. The calculator will determine if the patient it eligible or not using the criteria outlined below.

Note: All sepsis cases should be run through the organ function calculator if they do not have any of the above exclusion criteria.

Acute organ dysfunction is identified as any one of the following:

- Respiratory support greater than 4L (35%) oxygen for >2 hours on a given calendar day.
    - This does not need to be consecutive.
    - AND no ICD10 for chronic respiratory failure with hypoxemia (J96.11), acute on chronic respiratory failure (J96.21), or history of home oxygen use for respiratory cases (with acute respiratory failure, influenza, pneumonia or COVID-19 discharge ICD-10 code).

  INCLUDE: Increased Respiratory support also includes administration of mechanical ventilation, NIPPV, increased CPAP/BiPAP settings during days 1-2 of the hospital encounter.
- Serum Cr ≥1.2 AND 50% increase from baseline (lowest value during hospitalization) AND no ICD10 for end-stage renal dysfunction (N18.6) coded on admission.
- Platelet count < 100 cells/μL AND > 50% decline in platelets from baseline (highest value during hospitalization)
- Total bilirubin ≥ 2.0 mg/dL AND doubling of total bilirubin from baseline (lowest value during hospitalization)
- Lactate > 2.0 mmol/L
- Treatment with intravenous vasopressor (Angiotensin II, Dopamine, Epinephrine, Norepinephrine, Phenylephrine, or Vasopressin) outside of operating room during days 1-2 of the hospital encounter.
- Presentation of altered mental status
  INCLUDE: Confusion, reports that the patient is acting out of usual character, worsening deviation from baseline mental status, somnolence, comatose state, encephalopathy, unresponsiveness. Please also include Nursing documentation of altered mental status(es).

Abstraction Process

Once a case has been deemed eligible, it will then be routed for full abstraction of detailed clinical data. Each case will capture data from 90 days prior to the hospital admission through 90 days following the hospital discharge. A subset of forms will be required on all cases, and another subset of forms are to be used as they are applicable for the specific patient specific scenarios. Examples of optional forms include forms that capture diagnostic test results or discharge medications. After all of the applicable data entry forms are completed, a 90-Day Phone Call for patients who are alive and eligible for a phone call must be completed.

© 2026 Conlon A et al. *JAMA Network Open*.

eReference

1. Gupta AB, Heath M, Walzl E, et al. Antibiotic De-Escalation in Adults Hospitalized for Community-Onset Sepsis. *JAMA Intern Med*. 2026;186(2):192-202. doi:10.1001/jamainternmed.2025.6919

© 2026 Conlon A et al. *JAMA Network Open*.
