# Supplementary material for: Presenting Symptoms and Antibiotic Timing Among Hospitalized Adults With Presumed Sepsis
Source: JAMA Netw Open. 2026 Sep 28;9(9):e2635575. doi: 10.1001/jamanetworkopen.2026.35575 (PMC13621253; doi:10.1001/jamanetworkopen.2026.35575)
Supplement: Supplementary file 2 [file jamanetwopen-e2635575-s002.pdf]

Data Sharing Statement

Conlon. Patient Symptoms and Antibiotic Timing Among Hospitalized Adults Presenting With Sepsis. *JAMA Netw Open*. Published September 28, 2026.
doi:10.1001/jamanetworkopen.2026.35575

Data
Data available: No
